# Lipid homeostasis is essential for oogenesis and embryogenesis in the silkworm, *Bombyx mori*

**DOI:** 10.1007/s00018-024-05173-8

**Published:** 2024-03-12

**Authors:** Fangying Yang, Xiaoyan Xu, Bo Hu, Zhongjie Zhang, Kai Chen, Ye Yu, Hua Bai, Anjiang Tan

**Affiliations:** 1 Key Laboratory of Insect Developmental and Evolutionary Biology, CAS Center for Excellence in Molecular Plant Sciences, Shanghai Institute of Plant Physiology and Ecology, Chinese Academy of Sciences, Shanghai, 200032 China; 2 Jiangsu Key Laboratory of Sericultural Biology and Biotechnology, School of Biotechnology, Jiangsu University of Science and Technology, Zhenjiang, 212100 China; 3 Key Laboratory of Silkworm and Mulberry Genetic Improvement, Ministry of Agriculture and Rural Affairs, The Sericultural Research Institute, Chinese Academy of Agricultural Sciences, Zhenjiang, 212100 China; 4 Core Facility Center, CAS Center for Excellence in Molecular Plant Sciences, Chinese Academy of Sciences, Shanghai, 200032 China; 5 Department of Genetics, Development, and Cell Biology, Iowa State University, Ames, IA 50011 USA

**Keywords:** Bombyx mori, Lipid metabolism, Lipidomics, Fecundity

## Abstract

Reproduction, a fundamental feature of all known life, closely correlates with energy homeostasis. The control of synthesizing and mobilizing lipids are dynamic and well-organized processes to distribute lipid resources across tissues or generations. However, how lipid homeostasis is precisely coordinated during insect reproductive development is poorly understood. Here we describe the relations between energy metabolism and reproduction in the silkworm, *Bombyx mori*, a lepidopteran model insect, by using CRISPR/Cas9-mediated mutation analysis and comprehensively functional investigation on two major lipid lipases of Brummer (BmBmm) and hormone-sensitive lipase (BmHsl), and the sterol regulatory element binding protein (BmSrebp). BmBmm is a crucial regulator of lipolysis to maintain female fecundity by regulating the triglyceride (TG) storage among the midgut, the fat body, and the ovary. Lipidomics analysis reveals that defective lipolysis of females influences the composition of TG and other membrane lipids in the *BmBmm* mutant embryos. In contrast, BmHsl mediates embryonic development by controlling sterol metabolism rather than TG metabolism. Transcriptome analysis unveils that BmBmm deficiency significantly improves the expression of lipid synthesis-related genes including *BmSrebp* in the fat body. Subsequently, we identify BmSrebp as a key regulator of lipid accumulation in oocytes, which promotes oogenesis and cooperates with BmBmm to support the metabolic requirements of oocyte production. In summary, lipid homeostasis plays a vital role in supporting female reproductive success in silkworms.

**Supplementary Information:**

The online version contains supplementary material available at 10.1007/s00018-024-05173-8.

## Introduction

Reproduction success in living organisms is tightly associated with nutrition and energy metabolism [1–4]. Several human metabolic disorder diseases, such as lipodystrophy and insulin resistance, are generally accompanied by impaired fecundity [5, 6]. A growing number of evidence suggests that lipids accumulate in oocytes during oogenesis and energetically support reproductive success in *Drosophila melanogaster* [7–9]. However, the extent to which lipid metabolism is relevant to female fertility in non-*Drosophila* insect species is poorly understood. Understanding the mechanisms underlying lipid metabolisms-mediated oogenesis and embryogenesis will provide valuable insights into revealing how lipid homeostasis regulates insect fecundity and reproduction success.

In adipocytes, energy is stored in lipid droplets, whose cores are composed of triglycerides (TG) and sterol esters [10]. The breakdown of triglycerides requires a series of enzymatic reactions induced by triglyceride lipase (ATGL), hormone-sensitive lipase (HSL), and monoacylglycerol lipase (MGL) [11]. ATGL in mammalian cells is a major enzyme of lipolysis, catalyzing the initial step in TG hydrolysis [12]. ATGL orthologs in plants, yeast, and flies are identified as key regulators with similar lipid mobilization functions [13–15], highlighting their conserved role in TG hydrolysis. HSL acting in parallel and downstream of ATGL is mainly responsible for diacylglycerol catabolism [12, 16]. HSL exhibits broad substrate specificity and functions as a SE hydrolase in multiple tissues in mice [17–19]. However, both lipases show sexually different functions in reproduction success between mammals and insects. Ablation of ATGL in male mice induced lipid accumulation in testes and impaired male fertility [20]. In contrast, deficiency of the ATGL ortholog Brummer in the brown planthopper results in immaturity of oocyte and female infertility [21]. HSL knockout male mice are sterile because of oligospermia and increased amounts of cholesterol ester in testes [18]. *Drosophila* HSL specifically regulates sterol esters mobilization to promote intergenerational sterol transfer, which improves reproductive success in female flies [22].

Lipid homeostasis also relies on transcriptional regulation [23]. Transcription factors sense and transmit signals from different nutrient diets or hormones to regulate the enzymes involved in lipid metabolism. Sterol regulatory element binding protein (SREBP) is a membrane-bound transcription factor containing basal helix-loop-helix-leucine zip (bHLH-ZIP) structural domains anchored to the endoplasmic reticulum (ER) as inactive precursors [24]. The activation state of SREBP depends on the level of intracellular lipids. When the lipid level is low, the SREBP complex in ER transports to the Golgi membrane, where the protease cleaves the SREBP, and the released bHLH-Zip structural domain moves to the nucleus to activate transcription of genes to support lipid synthesis [25]. Recent studies have shown that *Drosophila* SREBP affects female reproduction by regulating lipid accumulation in oocytes [26]. However, whether SREBP contributes to reproduction success in other insect species remains unknown.

In the current study, we use the lepidopteran model insect, the domesticated silkworm, *Bombyx mori*, to investigate the association between lipid homeostasis and reproduction success through functional analysis focusing on two major lipid lipases BmBmm and BmHsl, and the transcription factor BmSrebp. Our findings revealed that BmBmm and BmSrebp orchestrate lipolysis and lipogenesis respectively to support *B. mori* female fecundity. BmHsl does not cooperate with BmBmm to regulate lipolysis. Instead, it promotes female reproductive success by regulating sterol metabolism. Our studies demonstrate the metabolic demands of oogenesis and embryogenesis, suggesting that TG and sterol ester metabolism are both essential for insect female fecundity.

## Results

### *BmBmm* is essential for lipid homeostasis in *B. mori*

**Fig. 1:**
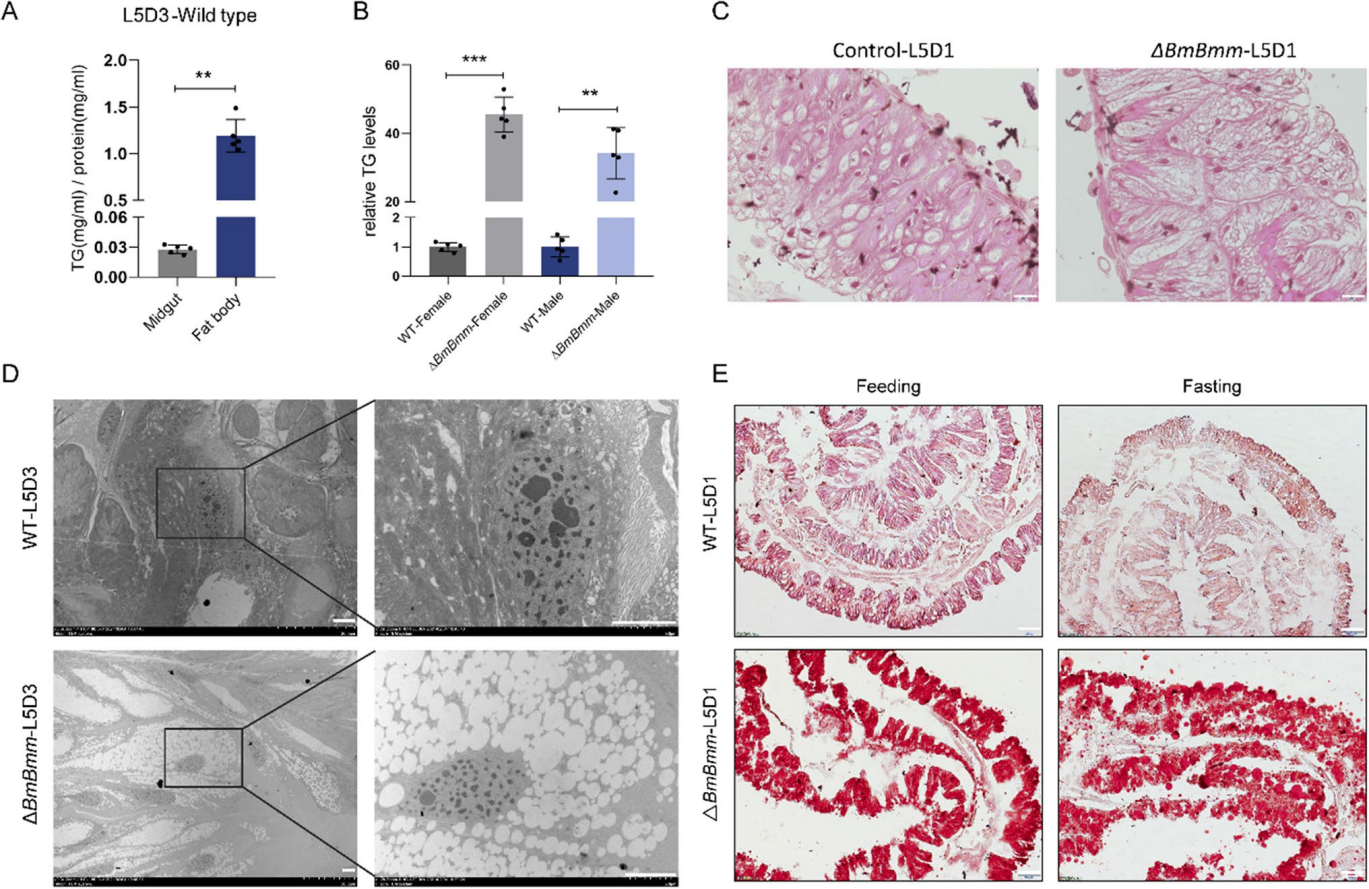
Excessive accumulation of lipid droplets in the midgut of *BmBmm* mutants. **A** Relative content of TG in the midgut and fat body from WT females (fifth instar, day 3) were determined in five biological replicates (each replicate contains tissue from three animals). **B** Relative TG levels in the midgut of female and male silkworms from WT and Δ*BmBmm* (fifth instar, day 3) were determined in five biological replicates (each replicate contains tissue from three animals). Data are normalized to WT. **C** The H&E staining of midgut, and tissues were taken from WT and Δ*BmBmm* females (fifth instar, day 3). Scale bar represents 50 μm. **D** TEM images of enterocytes in the midgut. Tissues were taken from WT and Δ*BmBmm* females on the 3rd day of the 5th instar. Scale bar represents 5 μm. **E** Oil red O-stained images of midgut. Samples were obtained from WT and Δ*BmBmm* females (fifth instar, day 1) and after 72 h of starvation. Scale bar represents 100 μm**.** Error bars represent means ± SDs. **p < 0.01; ***p < 0.001

A previous study reported that Bmm was highly enriched in energy storage organs and in the food-absorbing part of the digestive tract in *Drosophila* [15]. The relative mRNA level of *BmBmm* investigated by using qRT-PCR revealed that *BmBmm* was predominantly expressed in the midgut during the fifth larval instar (Figure S1B and S1C), indicating that *BmBmm* may play an important role in digesting process. Given the known impact of its homolog on lipolysis, BmBmm containing a patatin-like domain may regulate the TG hydrolysis in *B. mori* (Figure S1A). To better understand the physiological role of *BmBmm*, we used the CRISPR/Cas9 system to generate the *BmBmm* mutant (Δ*BmBmm*) (Figure S1D) and isolated homozygous deletion mutant (*BmBmm*^−/−^) (Figure S1E). Normally TG hardly accumulates in the larval midgut of wild-type (WT) silkworms (Fig. 1A). However, the amount of TG significantly increased in the midgut of *BmBmm* mutant larvae (Fig. 1B). Depletion of BmBmm led to a marked increase in lipid storage in the intestinal epithelium manifested by excess content and number of lipid droplets, revealed by H&E-stained histological images and electron-microscopic images investigation (Fig. 1C and D).

**Fig. 2:**
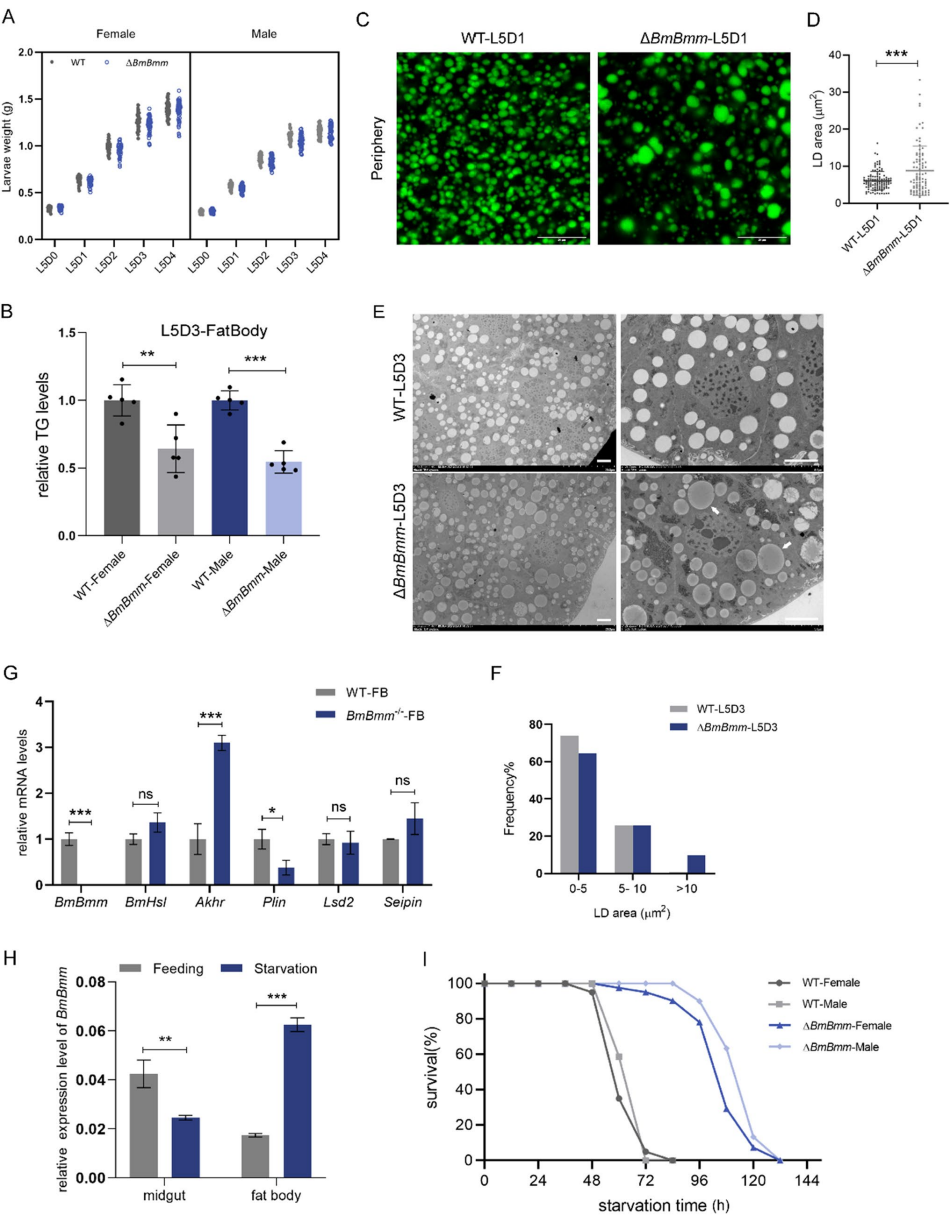
The absence of BmBmm affects lipid droplet size in the fat body and energy mobilization. **A** Body weight analysis of female and male 5th instar larvae from WT (n = 94) and Δ*BmBmm* (n = 100) per day. **B** Relative TG levels in the fat body of female and male silkworms from WT and Δ*BmBmm* (fifth instar, day 3) were determined in five biological replicates (each replicate contains tissue from three animals). Data are normalized to WT. **C** The lipid droplets of WT and Δ*BmBmm* females visualized by BODIPY staining of the fifth-instar larval fat body. Scale bar represents 20 μm. **D** Quantification of lipid droplet area in adipocytes in panel **C**. **E** TEM images of fat body taken from WT and Δ*BmBmm* females (fifth instar, day 3). Arrows indicate giant lipid droplets. Scale bar represents 5 μm. Quantitative analyses of lipid droplet areas are summarized in **F**. **G** Relative mRNA levels of lipid droplet size-regulating genes in the fat body from WT and *BmBmm*^−/−^ females were determined by qRT-PCR in three biological repeats (each replicate contains three silkworms). Data are normalized to WT. **H** Relative expression levels of *BmBmm* in the midgut and fat body before and after starvation treatment were determined in three biological replicates (each replicate contains three silkworms). **I** Survival statistics of larvae under starvation stress conditions. Females and males of WT and Δ*BmBmm* were starved on the 1st day of the fifth instar. Error bars represent means ± SDs. *p < 0.05; **p < 0.01; ***p < 0.001; *ns* non-significant

Larvae lacking BmBmm did not exhibit significant deviation in weight (Fig. 2A). Considering the over-abundance of TG in the Δ*BmBmm* midgut, we wondered about the effect of *BmBmm* mutation on the TG content in the fat body. A significant decrease was assessed in the Δ*BmBmm* fat body (Fig. 2B), implying that fat body may not obtain enough TG content from the midgut in the mutants. To explore whether defective lipolysis affects lipid size in fat body, we investigated lipid droplets visualized by the lipophilic BODIPY dye and using transmission electronic microscopy. The results showed that BmBmm deficiency induced the formation of giant lipid droplets (Fig. 2C and E), which were barely detected in age-matched WT animals (Fig. 2D and F). We subsequently assessed the expression level of several genes involved in regulation of lipid droplet size (Fig. 2G). *perilipin* (*plin*), whose homologous gene in *Drosophila* plays a role in regulating lipid droplet size [27], had a significantly lower mRNA level in mutants (Fig. 2G). Although IE_2_-driven *BmBmm* overexpression had no obvious effect on lipid droplets in wild-type fat body (Fig. S2B and S2C), it significantly alleviated lipid accumulation in the *BmBmm*^−/−^ midgut (Figure S2D). We speculated this was due to the low level of overexpression (Figure S2A).

The response to starvation stress can be used as one of the criteria for judging whether energy metabolism is disordered. As previously reported, starvation-induced consumption of TG was impaired in Bmm-deficient *Drosophila* [28]. Oil-red o-staining positive lipid droplets were still present in the midgut of *BmBmm* mutants after fasting, indicating that fat mobilization was impaired (Fig. 1E). In *B mori* larvae, the mRNA levels of *BmBmm* after starvation were significantly up-regulated in the fat body, whereas were significantly down-regulated in the midgut (Fig. 2H). The data displayed differential regulation of the response to starvation in the midgut and fat body of the silkworms. In addition, *BmBmm* mutants survived significantly longer than did WT animals after starvation (Fig. 2I). Altogether, these data indicate that BmBmm is responsible for TG mobilization in silkworm larvae.

### Decreased TG levels of embryos lead to reduced fecundity of *BmBmm* mutant females

**Fig. 3:**
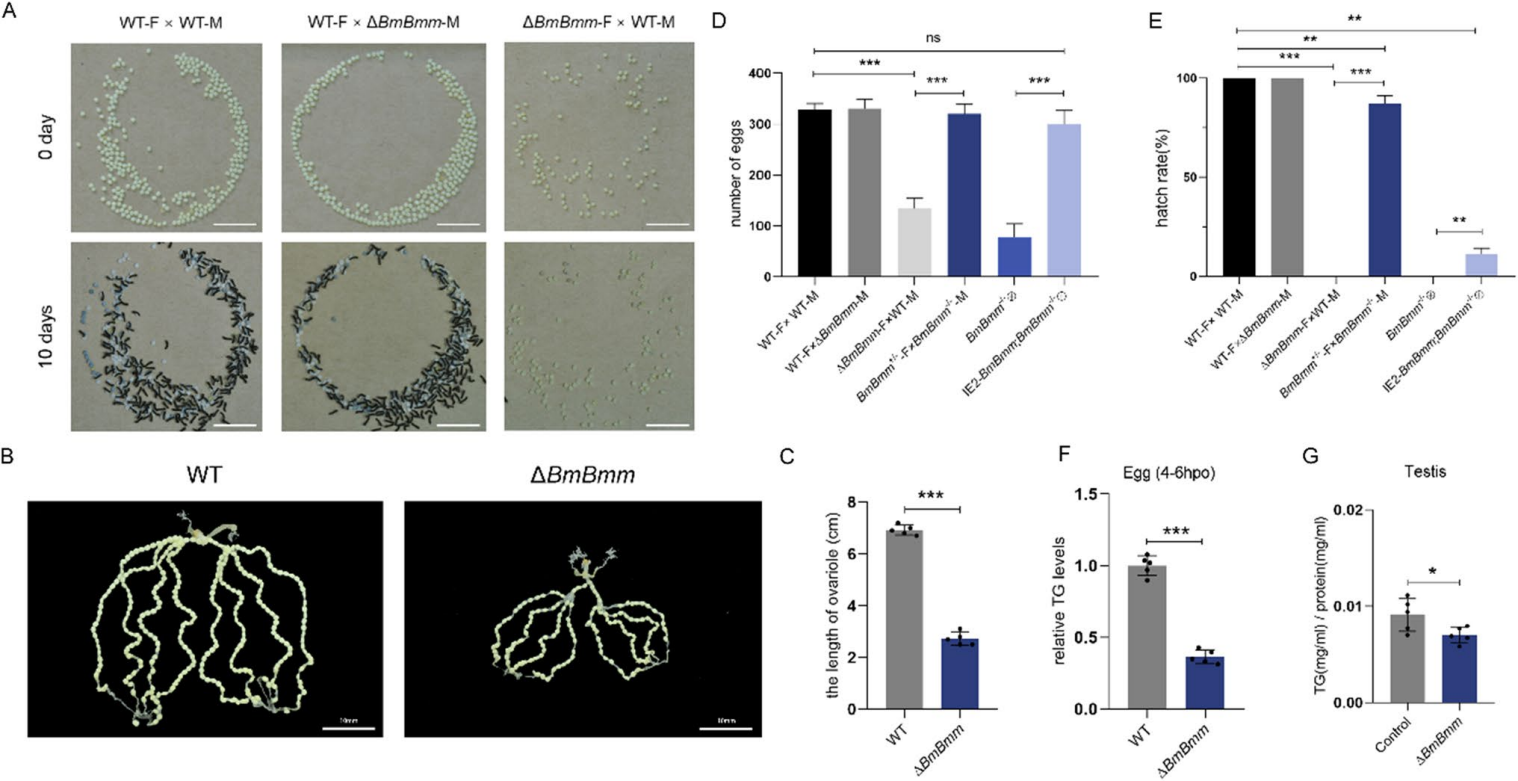
BmBmm deficiency leads to decreased fecundity in females. **A** Photographs of eggs laid by WT females mated with WT and Δ*BmBmm* males respectively and Δ*BmBmm* females mated with WT males. The eggs laid by Δ*BmBmm* females did not hatch on the 10th day. Scale bar represents 10 mm. **B** Photographs of ovarioles from WT and Δ*BmBmm*. Scale bar represents 10 mm. **C** The length of ovarioles. **D** The number of eggs laid by females within 24 h was counted (n = 10). Female is represented by F. Male is represented by M. **E** The hatching rates of eggs were analyzed after 10 days since the female moths laid eggs (n = 10). Female is represented by F. Male is represented by M. **F** Relative TG levels of silkworm eggs laid by female moths of WT and Δ*BmBmm* were determined in five biological replicates (each replicate contains tissue from three animals). Data are normalized to WT. **G** Relative content of TG of testis from WT and Δ*BmBmm* males on the 7th day of pupal stage were determined in five biological replicates (each replicate contains tissue from three animals). Error bars represent means ± SDs. *p < 0.05; **p < 0.01; ***p < 0.001; *ns* non-significant

To confirm the physiological relevance between TG metabolism and female fecundity, we investigated the quantity and quality of eggs produced by WT and Δ*BmBmm* females. The length of ovarioles was significantly reduced and had 60–75% fewer eggs in mutant female moths (Fig. 3A–D). In *Drosophila*, Bmm plays an essential role in embryogenesis, and embryonic lethality can be partially restored by zygotic effects [15]. However, embryos of F1 hybrid between Δ*BmBmm* females and WT males did not hatch (Fig. 3A and E) normally, indicating the fecundity of abnormal oocytes could not be partially reverted by a paternally provided functional *BmBmm* gene. In addition, the hatching rate of embryos produced by *BmBmm*^+/−^ females mated with *BmBmm*^−/−^ males was slightly lower than that of *BmBmm*^+/−^ females mated with WT males (Fig. 3E), suggesting that zygotic BmBmm loss has an impact on embryonic development. We also found that systemic overexpression of *BmBmm* can partially restore the reproductive-associated phenotype (Fig. 3D and E). These results suggest the infertility of BmBmm-deficient females is mainly caused by the maternal effect and zygotic BmBmm deficiency affects embryonic development.

Given the impaired TG mobilization in *BmBmm* mutants, we wondered if BmBmm deficiency may also impact the TG content of embryos. As expected, we found that TG levels of embryos laid by *BmBmm* mutant females changed quite dramatically, which reduced by approximately 60% (Fig. 3F). We also measured the TG content of the testes during the pupal stage. Although the TG of the mutant testes was lower than that of the WT, the testes contained almost no TG (Fig. 3G). Collectively, these results suggest a key role for BmBmm in female fecundity by controlling lipid levels in the whole body.

### Defective lipolysis influences the composition of TG and other membrane lipids in the embryo

**Fig. 4:**
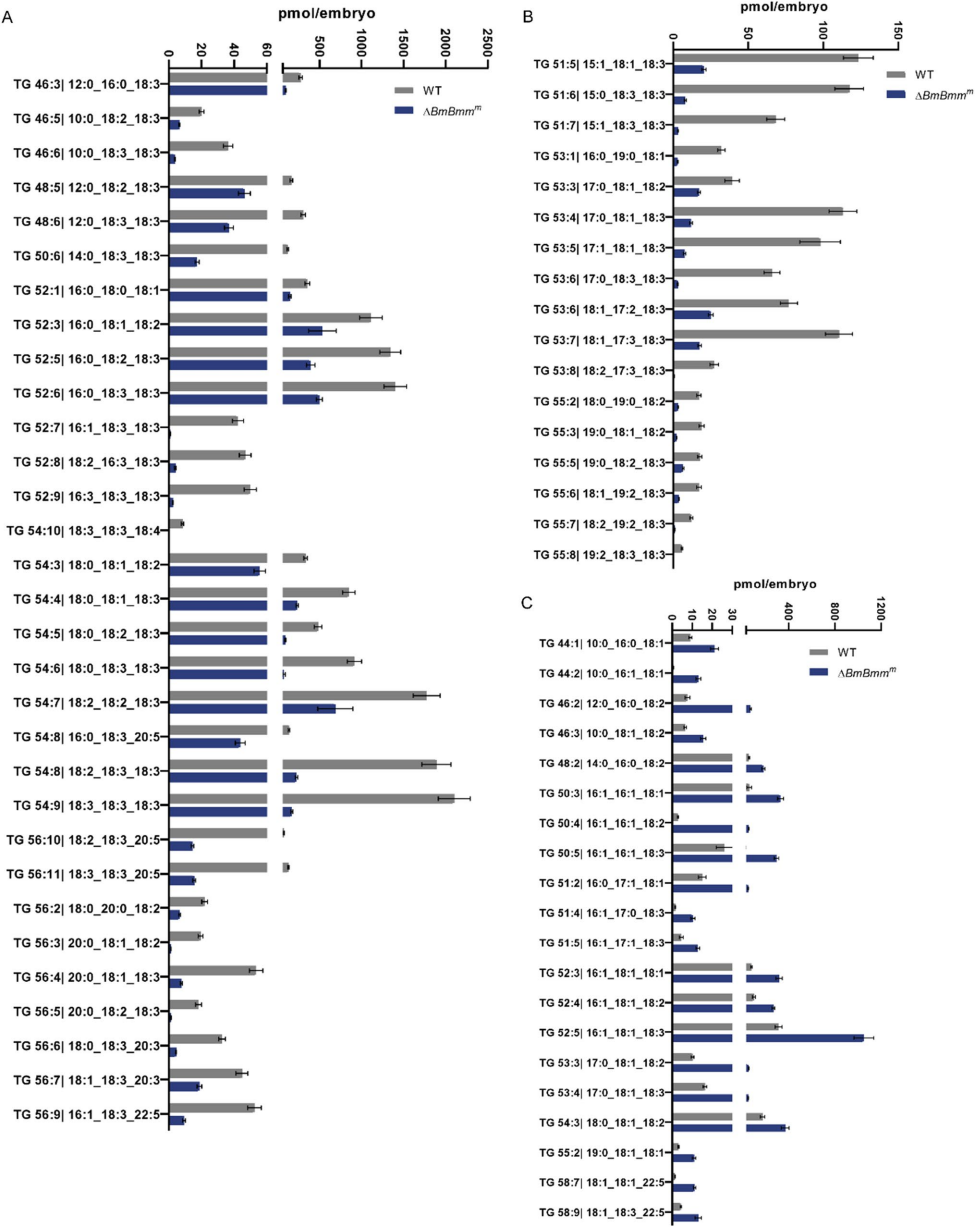
Comparison of the relative contents of glycerides in WT and Δ*BmBmm*^m^ embryos. **A** The relative content of triglyceride species with an even number of carbon atoms in silkworm eggs was significantly lower in Δ*BmBmm*^m^ than in WT (n = 5, Student’s t test, p < 0.05). **B** The relative content of triglyceride species with an odd number of carbon atoms in silkworm eggs was significantly lower in Δ*BmBmm*^m^ than in WT (n = 5, Student’s t test, p < 0.05). **C** Statistics of triglyceride species whose relative content in Δ*BmBmm*^m^ was significantly higher than that in WT (n = 5, Student’s t test, p < 0.05)

To understand the impact of maternal lipolysis disorders on the lipid composition in embryos, we monitored the lipidome of embryos laid by WT and mutant females (Δ*BmBmm*^m^) after mating with WT males at the initial stage of embryogenesis (7 h post-oviposition) respectively. Lipidomic analysis showed that the higher content of TG in the embryos of the WT group was mainly concentrated in the carbon chains with 54 and 52 carbon atoms (Fig. 4A). The most abundant TG in WT animals was TG 54:9 with three 18:3 fatty acid moieties, followed by TG 54:8 with two 18:3 and one 18:2 fatty acid branches (Fig. 4A), suggesting high unsaturation degree during the early embryonic stage. Compared with WT embryos, Δ*BmBmm*^m^ embryos contained more TG species with 16:1 fatty acid moiety but less triacylglycerol species with 18:3 fatty acid moiety (Fig. 4A–C). Therefore, Δ*BmBmm*^m^ exhibited less content of high unsaturated TG containing 5 or more double bonds (Fig. 4A–C). These results demonstrated that the absence of BmBmm caused a transition of the fatty acid composition of the TG moiety toward low unsaturated chain fatty acids in embryos, implying a higher saturation degree of TG. Consistently, Embryos of Δ*BmBmm*^m^ accumulated more diacylglycerol with two 16:1 fatty acid moieties and less diacylglycerol with two 18:3 fatty acid moieties (Figure S3A). As expected, the relative content of FA18:2 and FA18:3 in eggs laid by mutant females was significantly reduced, while FA16:1 was significantly increased (Figure S3B). Furthermore, we assessed levels of major glycerophospholipids including phosphatidylcholine (PC), phosphatidylethanolamine (PE), and phosphatidylinositol (PI). The results showed that the changing trend of PC species between WT and mutants was similar to that of TG (Figure S4A). Consistently, PC 36:6 with two 18:3 fatty acid moieties in BmBmm^m^ was 4.5-fold less abundant than WT (Figure S4A). The abundance of PC32:2 with two 16:1 fatty acid moieties in BmBmm^m^ was significantly higher than that of WT (Figure S4A). The change trend of the abundance of PE36:6 with two 18:3 fatty acid moieties and PE32:2 with two 16:1 fatty acid moieties in BmBmm^m^ was also similar to that of PC (Figure S4B). In addition to PI34:4, the relative content of other PIs with 18:3 fatty acid moiety was significantly reduced in BmBmm^m^ (Figure S3C). Taken together, these data suggest maternal BmBmm deletion not only affects the composition of TG but also induces remodeling of other membrane lipids in embryos.

### *BmHsl* mutation does not change TG levels

As HSL was a known lipolysis enzyme exhibiting broad substrate specificity in many species, we also investigated whether disrupted HSL signaling also affects lipid metabolism and fecundity in the silkworm. Similar to homologues in other species, BmHsl contained an N-terminal HSL_N domain and an abhydrolase domain (Figure S5A). We performed CRISPR/Cas9-mediated gene editing (Figure S5B) and *BmHsl*^−/−^ mutants with 1.179 kb genomic sequences deletion were obtained (Figure S5C). Unlike *BmBmm* mutants, *BmHsl* mutants did not change sizes and abundances of lipid droplets (Figure S6A and S6B). TG levels of fat body and midgut in female and male larvae did not show significant variation between mutant and WT animals (Figure S6C and S6D). To rule out that BmHsl acts in concert with other TG lipolysis pathway genes, we evaluated the expression of genes which have predicted function in TG mobilization. The mRNA level analyses showed no significant change in response to BmHsl deficiency in the fat body (Figure S6E). Also, the expression levels of fatty acid synthesis genes were similar in *BmHsl* mutant and control animals (Figure S6F). These data imply that BmBmm and BmHsl may be involved in different signaling pathways. Then, we measured survival rates in *BmHsl* mutant larvae during starvation. The survival ability showed no significant difference between WT and *BmHsl* mutants (Figure S6G). These data suggest BmHsl-deficient silkworms have normal TG and energy metabolism.

### BmHsl is essential for embryonic sterol metabolism rather than lipolysis to support reproduction

**Fig. 5:**
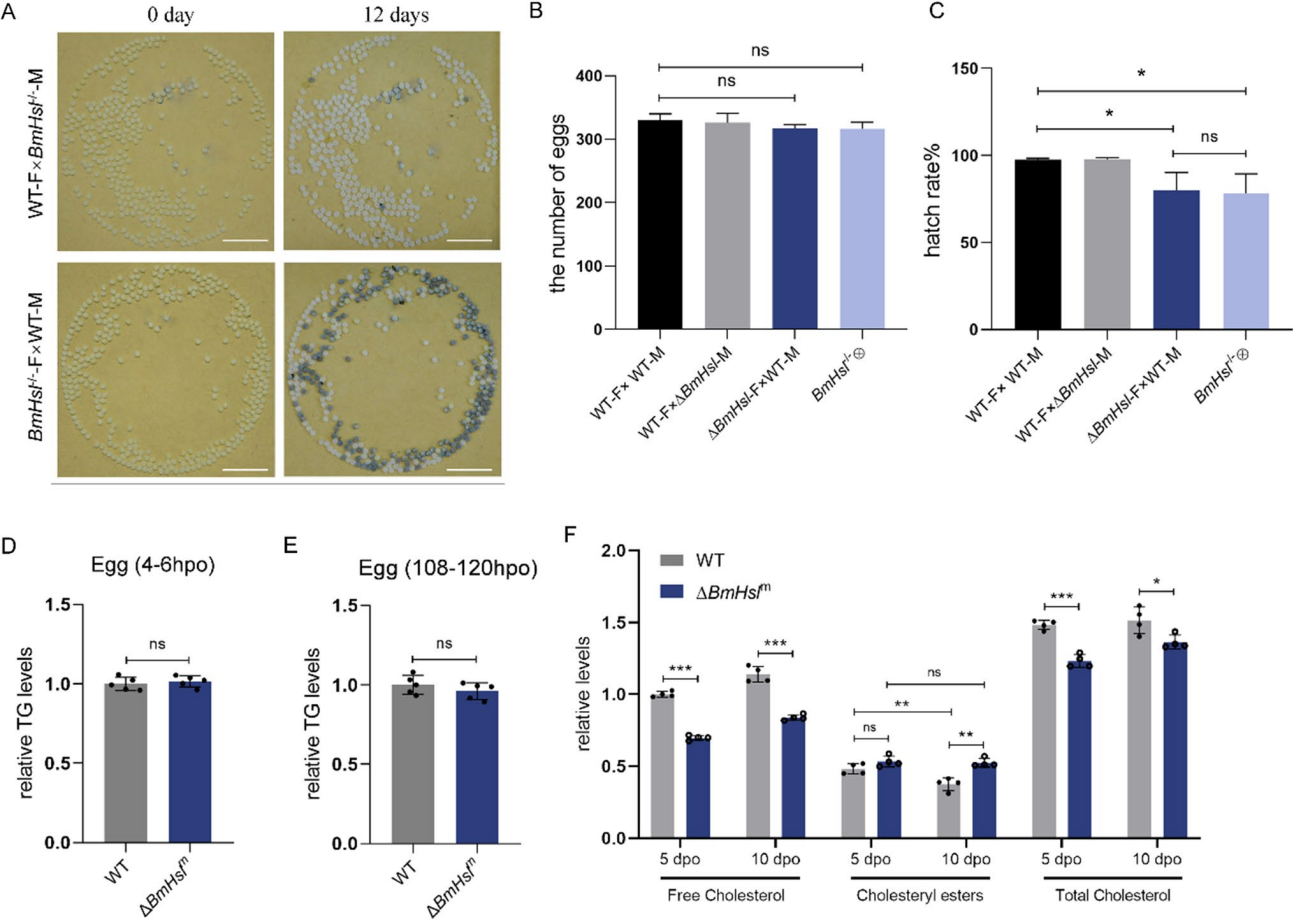
Maternal BmHsl affects embryonic cholesterol homeostasis. **A** Photographs of eggs laid by WT and BmHsl^−/−^ females mated with BmHsl^−/−^ and WT males respectively. A portion of eggs laid by BmHsl^−/−^ females did not hatch. Scale bar represents 10 mm. **B** The number of eggs laid by females within 24 h was counted (n = 10). Female is represented by F. Male is represented by M. **C** The hatching rates of eggs were analyzed after 10 days since the female moths laid eggs (n = 10). Female is represented by F. Male is represented by M. **D** and **E** Relative TG levels of silkworm embryos laid by WT and Δ*BmHsl* females (Δ*BmHsl*^m^) at 4–6 h (**D**) and 108–120 h (**E**) post-oviposition (hpo) were determined in five biological replicates (each replicate contains tissue from three animals). Data are normalized to WT. **F** Relative levels of free cholesterol, cholesteryl esters and total cholesterol of silkworm embryos laid by WT and Δ*BmHsl*^*m*^ females on 5th and 10th day post-oviposition (dpo) were determined in four biological replicates (each replicate contains tissue from three animals). Data are normalized to WT. Error bars represent means ± SDs. *p < 0.05; **p < 0.01; ***p < 0.001; *ns* non-significant

We subsequently investigated whether female fertility was affected by BmHsl deficiency. The number of eggs was comparable between *BmHsl*^*−/−*^ (or Δ*BmHsl*) and WT moths (Fig. 5A and B), whereas the hatchability of eggs laid by *BmHsl*^*−/−*^ (or Δ*BmHsl*) females crossed with WT males was lower than that of WT (Fig. 5A and C). On the contrary, embryos of F1 hybrids between WT females and *BmHsl*^*−/−*^ (or Δ*BmHsl*) males develop normally (Fig. 5A and C), suggesting that maternal BmHsl acted more importantly in embryogenesis. To determine whether BmHsl acts in embryo TG metabolism, we measured TG levels of embryos from WT females and Δ*BmHsl* females crossed with WT males during the early (4–6 h post-oviposition) and middle (108–120 h post-oviposition) stages and found that embryo TG levels were not significantly altered (Fig. 5D and E).

Previous studies showed HSL was responsible for catalyzing the hydrolysis of cholesteryl esters in multiple tissues of mice [17, 18]. Cholesterol, which is the main sterol, acts as a key component of cell membranes and biosynthesis of steroid hormones in insects [29, 30]. To determine the maternal effects of BmHsl in embryonic cholesteryl esters hydrolysis, we compared the levels of embryonic free and esterified cholesterol in heterozygous embryos (Δ*BmHsl*^*m*^) from BmHsl mutant females and WT males to the levels of WT embryos. The data showed that the levels of free cholesterol and total cholesterol were significantly reduced in embryos laid by BmHsl-deficient females (Fig. 5F). In contrast, Δ*BmHsl*^*m*^ embryos have significantly higher cholesteryl ester levels than WT embryos on 10th day (Fig. 5F). The cholesteryl ester content of WT embryos decreased significantly on day 10 compared to day 5, whereas there was no significant change in cholesteryl ester content in Δ*BmHsl*^*m*^ embryos (Fig. 5F). These findings imply a defective cholesteryl esters hydrolysis in Δ*BmHsl*^*m*^ embryos. To find out whether the cholesterol reduction was caused by abnormal expression of cholesterol metabolic genes influenced by *BmHsl* mutation, we assessed transcript levels of predicted sterol metabolic genes, including *NPC intracellular cholesterol transporter 1* (*Npc1*), *sterol O-acyltransferase 2* (*Soat*) and *3-hydroxy-3-methylglutaryl-CoA reductase* (*Hmgr*). Only a slight reduction of *Hmgr* mRNA levels in fat body of *BmHsl* mutants was observed (Figure S7A), whereas expression of other sterol metabolic genes showed no significant change in BmHsl-deficient fat body (Figure S7A).

Several non-esterified sterols contents of embryos were investigated by using liquid chromatography-tandem mass spectrometry (LC–MS/MS). The result showed that sterol composition in embryos was dominant by cholesterol, accompanied by low levels of sitosterol and ergosterol and trace amounts of stigmasterol (Figure S7B–S7E). Consistent with the free cholesterol levels determined by the colorimetric method, the Δ*BmHsl*^*m*^ embryos had lower cholesterol levels in the early and late phases of embryogenesis (Figure S7B). Besides, the content of ergosterol and stigmasterol were significantly reduced in embryos on day 9 post-ovulation (Figure S7C and S7E). Although we noticed a slight up-regulation of sitosterol in early embryogenesis of Δ*BmHsl*^*m*^, its levels did not change significantly in late embryogenesis (Figure S7D). Taken together, these results imply that different from BmBmm regulating lipolysis, BmHsl regulates embryonic development through sterol metabolism, especially cholesterol metabolism.

### The lipolysis deficiency promotes lipogenesis in the fat body

**Fig. 6:**
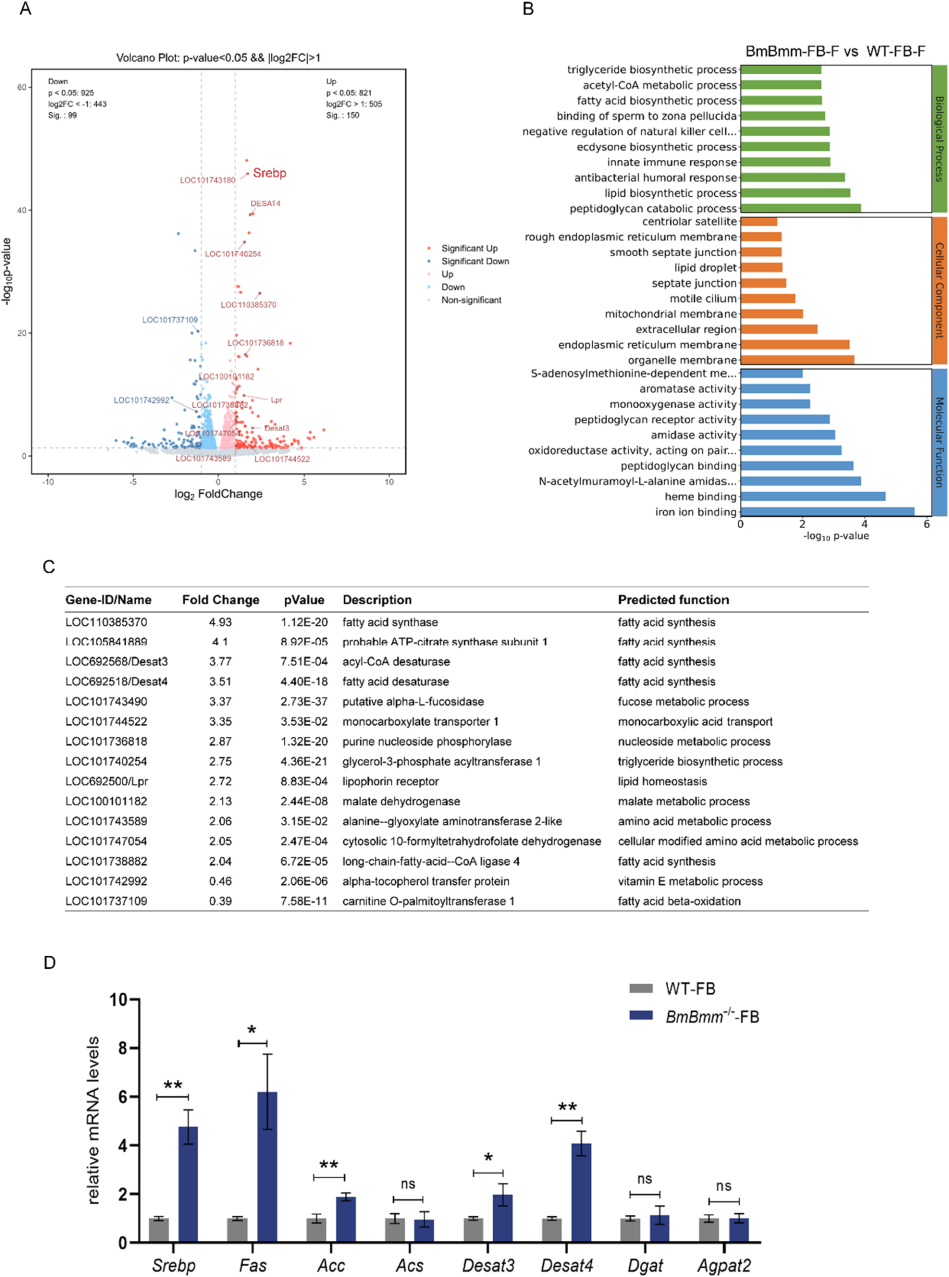
The lipogenesis signaling is stimulated in the fat body of Δ*BmBmm* females. **A** Volcano plot of differential genes from fat body of WT and Δ*BmBmm* females. **B** Top 30 of GO enrichment terms of up-regulated differential genes in fat body of Δ*BmBmm* females. **C** List of metabolic genes whose transcript levels were significantly changed in fat body of Δ*BmBmm* females in RNA-Seq. **D** Relative mRNA levels of lipid metabolism-related genes in the fat body from WT and *BmBmm*^−/−^ females were determined by qRT-PCR in three biological repeats (each replicate contains three silkworms). Data are normalized to WT. Error bars represent means ± SDs. *p < 0.05; **p < 0.01; *ns* non-significant

Adult insects that do not feed rely on energy reserves accumulated during the larval feeding stages to support life and reproduction [31]. The fat body, as an important transit station for energy storage and utilization, was gradually dissociated to support life and reproduction during the pupal stage of the silkworm. BmBmm-deficient females have more severe reproductive impairment than BmHsl-deficient females. To explore the signaling mechanisms cooperating with BmBmm to promote female fecundity, we conducted transcriptome sequencing analysis on the fat body of WT and *BmBmm* mutants. 99 genes were downregulated and 150 genes were upregulated at least twofold in the BmBmm deficient fat body on the first day of pupa (Fig. 6A). Top10 biological processes in GO enrichment analysis showed that the up-regulated differential genes were mainly involved in triglyceride biosynthetic pathway, acetyl-CoA metabolic process, fatty acid biosynthetic pathway, and lipid biosynthetic pathway (Fig. 6B). Among these DEGs, 15 predicted metabolic genes significantly changed and 8 of these genes have predicted functions in lipid metabolism (Fig. 6C). Importantly, lacking BmBmm remarkably induced the transcript level of genes involved in fatty acid synthesis, such as *fatty acid synthase* (*Fas*), *probable ATP-citrate synthase subunit 1*, *acyl-CoA desaturase* (*Desat3*), *fatty acid desaturase* (*Desat4*), *long-chain-fatty-acid–CoA ligase 4* in the fat body (Fig. 6C). We next verified the expression changes of several genes involved in lipogenesis process by qRT-PCR, including *Fas*, *acetyl-CoA carboxylase* (*Acc*), *acetyl-coenzyme A synthetase* (*Acs*), *Desat3*, *Desat4*, *diacylglycerol O-acyltransferase 1* (*Dgat*) and *1-acylglycerol-3-phosphate O-acyltransferase 2* (*Agpat2*). The mRNA concentrations of *Fas*, *Acc*, *Desat3* and *Desat4* were significantly up-regulated in response to BmBmm deficiency (Fig. 6D). Interestingly, we noticed the expression of *Srebp* displayed a significant increase in the fat body of mutants (Fig. 6D). Previous studies have shown that the transcription factor Srebp regulates lipid synthesis [25]. These results suggest that the defective lipolysis process stimulates lipogenesis signaling in the lipid-deficient fat body.

### BmSrebp signaling plays a central role in oogenesis

**Fig. 7:**
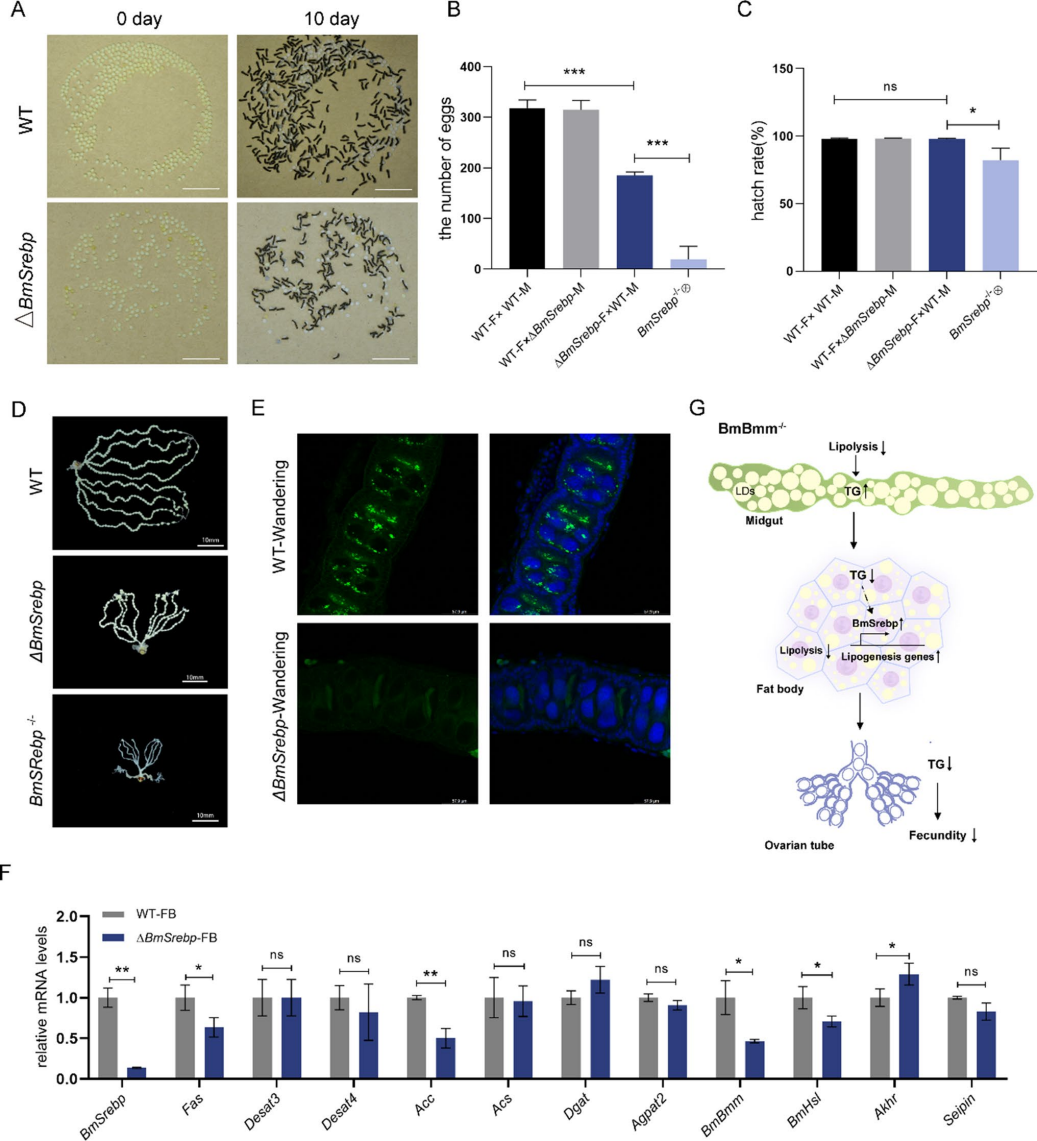
BmSrebp may support female fecundity by regulating lipid synthesis. **A** Photographs of eggs laid by WT and Δ*BmSrebp* females mated with WT males respectively. The eggs laid by Δ*BmSrebp* females can normally hatch. Scale bar represents 10 mm. **B** The number of eggs laid by females within 24 h was counted (n = 10). Female is represented by F. Male is represented by M. **C** The hatching rates of embryos were analyzed after 10 days since the female moths laid eggs (n = 10). Female is represented by F. Male is represented by M. **D** Photographs of ovarioles from WT and Δ*BmSrebp*. Scale bar represents 10 mm. **E** Immunofluorescent staining of oocytes in ovariole from WT and Δ*BmSrebp* females at the wandering stage. Cell nuclei were stained with Hoechst, shown in blue; lipid droplets were stained with BODIPY, shown in green. Scale bar represents 57.9 μm. **F** Relative mRNA levels of lipid metabolism-related genes in the fat body from WT and Δ*BmSrebp* females were determined by qRT-PCR in three biological repeats (each replicate contains three silkworms). Data are normalized to WT. **G** Putative model for the role of BmSrebp and BmBmm in lipid homeostasis and oogenesis. Error bars represent means ± SDs. *p < 0.05; **p < 0.01; *ns* non-significant

SREBP is a key transcription factor in the regulation of lipid homeostasis. In *Drosophila*, the inactivation of Srebp causes the deficiency of lipid droplets in oocyte [26]. Combined with the above result that *BmSrebp* mRNA levels were upregulated in the fat body of *BmBmm* mutants, we hypothesized that BmSrebp may play a role in female reproduction in silkworm. To understand the biological function of BmSrebp, we used a binary transgenic CRISPR/Cas9 system to generate *BmSrebp* mutants (Δ*BmSrebp*) and obtained homozygous mutants (*BmSrebp*^*−/−*^) with premature termination of translation (Figure S8A). The relative TG levels of midgut and fat body in mutants were similar with age-matched WT larvae (Figure S8D and S8E). Consistently, investigation of *BmSrebp* mutant fat body revealed no significant effect on size or abundance of lipid droplets (Figure S8B and S8C). However, BmSrebp deficiency in females significantly reduced the egg production (Fig. 7A, B and D), and homozygous mutant females produced very few to no eggs (Fig. 7D). The hatching rate of eggs laid by Δ*BmSrebp* females unchanged as compared to WT (Fig. 7A and C). We investigated the effect of BmSrebp deficiency on germline lipid accumulation at wandering stage. The immune-fluorescence staining images showed lipid storage defects in oocytes of mutant females (Fig. 7E). Then, we assessed the expression levels of genes involved in lipogenesis in fat body. Several known fatty acid synthesis genes, such as *Fas* and *Acc*, displayed decreased expression under *BmSrebp* mutation (Fig. 7F). Additionally, the mRNA concentration of *BmBmm* was significantly down-regulated in response to *BmSrebp* mutation (Fig. 7F). Taken together, we speculate that BmSrebp may promote oogenesis by regulating lipid synthesis and had a feedback loop with BmBmm (Fig. 7G).

## Discussion

In the current study, we performed a comprehensive analysis to investigate physiological functions of two major lipid metabolic enzymes BmBmm and BmHSL, and the transcription factor BmSrebp in the lepidopteran model insect *B. mori*. We present the first in vivo evidence that these three genes are essential to lipid homeostasis and female reproduction success in the silkworm.

Mammalian ATGL and its insect ortholog Brummer belong to patatin-like phospholipase domain-containing proteins including the lipase consensus sequence (Gly-X-Ser-X-Gly) [12, 15], are major lipases for the initiation of TG catabolism [12, 15, 32]. BmBmm deficiency resulted in accumulation of excessive TG in the intestinal epithelium of the larval midgut. Furthermore, BmBmm disruption induced formation of supersized lipid droplets, consistent with *Drosophila* [15]. Loss of Bmm activity causes obesity in flies [15], whereas we found the reduction of TG content in the fat body of *BmBmm* mutants. Meanwhile, the mRNA levels of genes related to fatty acid synthesis and de novo glyceride synthesis were significantly upregulated in the fat body of female mutants. We propose that defective lipolysis in the midgut may result in lipid deficiency in the fat body, which is similar to lipodystrophy. In general, these findings suggest that BmBmm is a functionally conserved lipolysis regulator.

In *Drosophila*, lacking both maternal and zygotic Bmm activity causes embryonic lethality and embryos produced by Bmm-deficient female flies with WT males hatched nearly 40% [15]. Overexpression of *Bmm* robustly increased fecundity in female flies [33] and accumulation of lipids in ovary is essential for oogenesis [7]. Our results showed that loss-of-BmBmm activity caused reduced egg production and complete infertility in female silkworms due to TG deficiency. Although loss of BmBmm resulted in excessive lipid accumulation in the midgut, lipids were deficient for other energy-demanding tissues. As a central energy depot, fat body not only accumulates and stores lipids, but also provides energy for various physiological activities through lipolysis [34–36]. We believe that the absence of BmBmm resulted in reduced lipid flow to the ovary during oogenesis. Our data also unraveled defective lipolysis changes in TG and other lipid compositions during the early stages of silkworm embryogenesis. Since most embryos laid by mutant females began to develop a shriveled phenotype at 24 h post-oviposition, we did not examine the lipidome at later stages of embryogenesis. The relative abundance of TGs, composed of polyunsaturated fatty acids, decreased in eggs laid by the mutant females. In mice, ATGL preferentially hydrolyzes unsaturated FAs [37, 38]. Similarly, these changes likely reflect the evolutionarily conserved function of BmBmm in preferential hydrolysis of high unsaturated level fatty acids. Together, our data highlight that BmBmm is an essential part of the lipid transport pathway among intestinal epithelium, adipocytes, and follicles.

HSL and ATGL coordinately mobilize stored TG in mammalian adipocytes [12]. Additionally, in adipose tissue and muscle, HSL also acts as principal enzyme for the cellular catabolism of diacylglycerol [39]. The deficiency of adipose HSL induces age-dependent liver steatosis, progressive lipodystrophy, and insulin resistance in mice [40]. In the third instar larvae of *Drosophila*, dHSL was a key regulator of acylglycerol metabolism [27]. The transcription level of *dHSL* was significantly increased in response to starvation stress [27]. However, unlike *BmBmm* mutants, the BmHsl-deficient silkworms did not display excessive lipid accumulation in *B. mori*, which was a common sign of defective lipolysis. Also, the response to starvation treatment, which was manifested by assessment of survival rate, was indistinguishable between *BmHsl* mutants and WT. BmHsl deficiency did not cause transcriptional changes in lipolysis, lipid storage-related and fatty acid synthesis genes. Our study raised questions about the role of BmHsl in glyceride metabolism in the silkworm. Accordingly, the lower egg hatching rate was not caused by TG content. Maternal BmHsl deficiency caused impaired cholesteryl ester hydrolysis in embryos. Our data imply a principal and conserved function of HSL in hydrolysis of SE. The mobilization of maternal sterol ester reserves depending on HSL is essential for egg production, which provides embryo with free sterols [22]. We speculate that the decrease in free cholesterol content in Δ*BmHsl*^*m*^ embryos is due to a reduction of cholesteryl ester allocation to embryos caused by a defect in maternal cholesteryl ester hydrolysis. The reduced fecundity in the silkworm may correlate with reduced embryonic free sterol levels. Given the mild phenotypes on egg laying in *BmHsl* mutants, there may be other important genes involved in sterol ester hydrolysis and mobilization into eggs, which deserves further exploration.

The delicate balance between lipolysis and lipogenesis is a key point in maintaining lipid metabolic homeostasis. In mammals, SREBP-1c was activated to promote fatty acid biosynthesis when unsaturated fatty acids (UFAs) are in short supply [41]. We observed a threefold increase in *BmSrebp* transcription level in the fat body of *BmBmm* mutants. Additionally, the expression of main genes involved in fatty acid synthesis also displayed a significant increase in response to BmBmm-deficiency. Previous studies have shown that SREBPs respond to nutrient cues and activate genes required for synthesis of fatty acid, phospholipid, TG and cholesterol, which contribute to regulating lipid homeostasis [42, 43]. As expected, the mRNA levels of *Fas* and *Acc* are down-regulated largely in *BmSrebp* mutants. This suggested that impaired TG hydrolysis in *BmBmm* mutants may lead to insufficient UFAs in the fat body, thereby feedback-regulating BmSrebp to promote fat synthesis signaling. In Drosophila, dietary supplementation with fatty acids rescues mutant larvae that die before 3rd instar to adulthood [44]. Since the silkworm feeds on mulberry leaves which naturally contain enough nutrients including fatty acids [45], we presume this is why the *BmSrebp* mutation does not affect fat body TG and supports the survival of the mutants. Interestingly, BmSrebp deficiency resulted in decreased *BmBmm* transcript levels. Recent evidence suggested that SREBP controls lipid levels in the germline to support reproduction of *Drosophila* [26]. Deletion of BmSrebp results in a reduction of lipid droplets in oocytes during early oogenesis. The number of eggs laid by *BmSrebp* mutant females was significantly reduced similar to the fecundity defects induced by *BmBmm* mutation. Although it is uncertain whether BmSrebp can physically interact with BmBmm, the present results indicated at least a feedback regulation between these two genes. Our data suggest that the reduced fecundity associated with disrupting BmSrebp function arises from the role it plays in regulating lipogenesis.

Increasing evidence shows tight correlations exist between lipid metabolism and reproduction in various organisms. Here we provide genetic evidence that BmBmm, as a TG lipase, is a key factor in regulating silkworm lipid homeostasis and female reproduction success. Our results also reveal that BmBmm collaborates with BmSrebp to regulate silkworm oogenesis. Furthermore, we also demonstrate that BmHsl plays a key role in cholesterol metabolism, not in lipolysis cooperating with BmBmm. Our work reveals the important role of energy homeostasis in the inter-tissue communication among the midgut, fat body and ovary, providing insights into understanding the relationships between energy homeostasis and reproduction in insects.

## Methods

### Silkworm strains and feeding conditions

Nistari, which is a multivoltine and non-diapause silkworm strain, was used in this study. All silkworm larvae were fed with mulberry leaves at 25 °C with a 12-h light/12-h dark cycle. The pupae and eggs were incubated at 25 °C.

### RNA extraction and quantitative real-time PCR

Total RNA was extracted from tissues using TRIzol Reagent (Invitrogen). The appropriate amount of samples was collected and homogenized in 1 mL TRIzol Reagent, followed by incubating at room temperature for 5 min. 200 μL chloroform was added to induce phase separation. The mixture was shaken vigorously, and centrifuged at 12,000×*g* for 15 min at 4 °C. The top aqueous layer was transferred and total RNA was precipitated by adding 500 μL isopropyl alcohol and 10 min incubation at room temperature. The mixture was centrifuged at 12,000×*g* for 10 min at 4 °C. Supernatant was removed and pellet was washed with 1 mL 75% ethanol, followed by centrifugation. This step was repeated three times. The pellet was dried and dissolved in RNAse-free water. cDNA was synthesized by using PrimeScript™ RT reagent Kit with gDNA Eraser (Takara).

qRT-PCR was performed on Bio-Rad CFX by using SYBR^®^Green Realtime PCR Master Mix (Toyobo). The thermal program for qRT-PCR was as follows: samples were held at 95 for 3 min, followed by 40 cycles of denaturation at 95 °C for 15 s, annealing at 60 °C for 15 s and extension at 72 °C for 20 s. Relative mRNA expression levels were determined by using *B. mori ribosomal protein 49* (*Bmrp49*) as normalization control.

### Generation of transgenic and mutant silkworms

Transgenic silkworms expressing Cas9, sgRNA and BmBmm were generated by piggyBac-mediated germline transformation. Cas9 plasmid (IE1-EGFP/nos-Cas9), sgRNA plasmid (IE1-DsRed/U6-sgRNA) and OE-*BmBmm* plasmid (IE1-DsRed/IE2-BmBmm) were individually added to the mixture of helper plasmids and *piggyBac* transposon mRNA and then separately microinjected into the fertilized egg laid within 6 h. The CRISPR/Cas9-based strategy was applied to disrupt *BmBmm*, *BmHsl* and *BmSrebp*. The nos-Cas9 strains and the U6-sgRNA strains were crossed to generate *BmBmm*, *BmHsl* and *BmSrebp* mutants (G2) with both EGFP and DsRed markers. Heterozygous mutants were screened from the offspring of mutants crosses with wild-type silkworms. Then heterozygous mutants were selfed to generate homozygous mutants. Genotyping was performed from moths by PCR to identify the mutation of targeted genes and those carrying the IE2-BmBmm transgene (TransDirect^®^ Animal Tissue PCR Kit).

### Immunohistochemistry and imaging analysis

For immunofluorescence staining of adipocytes and enterocytes, abdominal fat body tissue and mid-gut were dissected in PBS, fixed in 4% paraformaldehyde, followed by permeabilization in PBS containing 0.1% Triton X-100 for 10 min. Tissues were then incubated for 30 min in a 1:1000 dilution with PBS of 2 mg/mL BODIPY 493/503 (Invitrogen) and 5 mg/mL Hoechst. After transferring to glass slides, stained tissues are mounted in PBS containing 75% glycerol for microscopy analysis. Fluorescence images were captured by a laser scanning confocal microscope with × 100 1.4 numerical aperture (NA) oil objective lens (Olympus FV1000, Tokyo, Japan).

For immunohistochemical analysis of intestinal tissues, midgut was isolated and fixed in 4% paraformaldehyde. Tissues were dehydrated with ascending concentrations of sucrose solution at 4 °C. Then, samples were mounted in an OCT embedding compound, and frozen at − 20 °C. The frozen tissue blocks were cut into a 10 µm thickness using the cryostat (Leica CM1860). The tissue sections were dried for 30 min on a slide warmer at 37 °C. The slices were rinsed with 60% isopropanol and then stained with freshly prepared Oil Red O working solution for 15 min. After rinsed with 60% isopropanol and distilled water sequentially, samples were mounted in glycerine jelly. Microscopic images were taken by an upright microscope (Olympus BX51, Japan).

### Physiological studies

For body mass measurements, larvae were supplemented with enough fresh mulberry leaves. Beginning from the fourth molt (set as L5D0), larvae were weighed individually every 24 h for 5 consecutive days on an electronic analytical balance with a readability of 0.001 g (Sartorius). For the life span experiments under starvation condition, after the fourth molt (set as 0 h), three batches of 50 larvae for each genotype with an equal female/male ratio (1:1) were deprived of food. The survival rates were determined by regularly counting the number of surviving larvae. 72 h starved larvae were dissected and stained with BODIPY for analysis of lipid droplets. For fertility assays, 8 biological replicates per genotype were tested. The number of eggs was determined by counting the eggs laid by female mated with male for 4 h. The hatching rate (%) was determined as the total percentage of eggs that hatch from the total number of oviposited eggs.

### Triacylglycerol, free cholesterol and cholesteryl esters levels measurement

The determination of tissue TG levels was performed according to Tennessen et al. Tissues were frozen in liquid nitrogen and stored at − 80 °C. Frozen tissues were rapidly homogenized in PBST (PBS with 0.5% Tween 20) on ice. For TG level measurement, homogenized samples were incubated at 70 °C for 10 min. The TG in the sample was digested by Triglyceride Reagent (T2449, Sigma-Aldrich) at 37 °C for 1 h. The free glycerol was determined using the Free Glycerol Reagent (F6428, Sigma-Aldrich). The TG concentration was determined by subtracting the concentration of free glycerol in the PBST-treated samples from the total glycerol concentration in samples incubated with the Triglyceride Reagent. Homogenate without heat treatment was transferred to a new tube and then centrifuged at 13,000×*g* for 10 min at 4 °C. Protein content was measured from the supernatants by Bradford assay (Bio-Rad). The corresponding concentrations of triglycerides and proteins were calculated for each sample by conversion to the standard curve. TG levels were normalized to protein concentration in tissue sample. For free cholesterol and cholesteryl esters levels of embryos, lipids were extracted with 200 µL of chloroform: isopropanol: IGEPAL CA-630 (7:11:0.1), the content of free cholesterol and cholesteryl esters was measured using Cholesterol Quantitation Kit (MAK043, Sigma-Aldrich).

### Transmission electron microscope

Fat body and midgut dissected from silkworms were fixed with 2.5% glutaraldehyde and 0.2 M phosphate (pH 7.2) at 4 °C. Samples were rinsed in 0.1 M phosphate buffer, postfixed with 1% Osmium tetroxide for 2–6 h. After washed with 0.1 M phosphate, samples were dehydrated with ascending concentrations of ethanol, followed by adding 100% acetone. Samples were slowly infiltrated with 1 mL of resin: acetone mixture with different volume ratios and embedded into Epon812. After embedding with pure resin, samples were placed in oven for polymerization. Ultrathin sections (~ 50 nm) were stained with 2% uranyl acetate (15 min) and 10 mM lead citrate. The thin sections were examined by Hitachi HT7700 transmission electron microscopy. The areas of lipid droplet were analyzed by using ImageJ software.

### RNA-sequencing analysis

Triplicate samples of fat body and ovary were dissected from females of each genotype on the first day of pupa. Total RNA was extracted using an extraction kit (AM1561, Invitrogen). RNA quality was assessed by Bioanalyzer (Agilent) to verify RNA integrity and cDNA libraries were constructed using TruSeq Stranded mRNA LT Sample Prep Kit according to Illumina’s protocols. The libraries were sequenced using an Illumina HiSeq X Ten platform. For transcriptome resequencing, mRNA was isolated using DNase I and Oligo (dT), fragmented and used as templates of cDNA synthesized. Then, the cDNA was purified and treated with end reparation, single nucleotide A (adenine) addition and adapters addition. The suitable fragments were selected for the PCR amplification. After established, the sample library was sequenced using an Illumina HiSeq 2000 platform. Clean reads were mapped to the reference silkworm genome database (ASM15162V1, NCBI) using HISAT2 after raw reads were filtered (OE Biotech Co., Ltd). FPKMs were calculated using Cufflinks and the read counts were obtained by HTSeq-count. Differentially expressed genes (DEGs) were screened using the DESeq (2012) R package with P value < 0.05 and the absolute value of log2(foldchange) ≥ 1.

### Lipid extraction and lipidomic analysis

The analyses of lipidomic analysis were performed twice with 5 biological replicates. Each sample of 200 embryos was added 800 μL water and homogenized with a mental bead. Lipids in each sample were extracted by adding 3 mL methanol/dichloromethane (2:1) mixed solution containing 1:1000 diluted SPLASH lipidomix internal standard mix (Avanti Polar Lipids) and vortexed 1 h at room temperature. After the addition of 1 mL dichloromethane and 1 mL water to the samples in turn, samples were vortexed vigorously. Phase separation was induced by centrifugation at 12,000×*g* for 10 min. The lower organic phase was transferred to a glass vial and dried under a stream of nitrogen gas. Samples were reconstituted in 800 µL dichloromethane/isopropanol/acetonitrile/water (20:65:35:5, v/v/v/v) for lipidome measurements.

Samples were analyzed by Q Exactive quadrupole orbitrap high-resolution mass spectrometry coupled with a Dionex Ultimate 3000 RSLC (HPG) ultra-performance liquid chromatography (UPLC-Q-Orbitrap-HRMS) system (Thermo Fisher Scientific), with a HESI ionization source. 2 µL (positive) or 5 µL (negative) of samples was injected onto an acclaim C30 column (150 mm × 2.1 mm, 3 μm particle size; Thermo Fisher Scientific). The mobile phase consisted of mobile water: acetonitrile (40:60, v/v, A) and isopropanol: acetonitrile (90:10, v/v, B) both added 2 mM ammonium formate. The lipids were separated with an optimized gradient elution: 0–2.0 min, 30–43% B; 2.1–12.0 min, 55–65% B; 12.0–18.0 min, 65–85% B; 18.0–20.0 min, 85–100% B; 20.0–25.0 min, 100% B; 25.1–28.0 min, 30% B. The flow rate was 0.26 mL/min and the column temperature was 50 °C. All MS experiments were performed in positive and negative ion modes using a heated ESI source. The source and ion transfer parameters applied were as followed: spray voltage 3.5 kV (positive) and 2.8 kV (negative). For the ionization mode, the sheath gas, aux gas, capillary temperature, and heater temperature were maintained at 40, 10 (arbitrary units), 275 °C, and 350 °C, respectively. The S-Lens RF level was set at 50. The Orbitrap mass analyzer was operated at a resolving power of 70,000 in full-scan mode (scan range: 200–1800 m/z; automatic gain control (AGC) target: 1e6) and of 17,500 in the Top 10 data-dependent MS2 mode (stepped normalized collision energy: 20 and 35 for positive, 25 and 35 for negative; injection time: 80 ms; isolation window: 1.2 m/z; AGC target: 1e5) with a dynamic exclusion setting of 6.0 s.

### LC–MS/MS analysis of free sterol

The analyses of free sterol were performed twice with 5 biological replicates. A total of 100 embryos were homogenized in 200 µL isopropanol. The homogenate was dried under a stream of nitrogen gas. 2 mL MTBE, 0.6 mL MeOH and 0.6 mL H_2_O were successively added to each sample and vortexed for 20 min. Phase separation was induced by centrifugation at 4500 rpm for 10 min. The organic phase was collected and dried under a nitrogen stream. The dried samples were reconstituted in 50 µL methanol for instrumental analysis. Analysis of free sterol was conducted on a 6500 Plus QTRAP mass spectrometer (Sciex) with Exion-UPLC. The identity of the free sterols was determined by comparing retention time with standard of cholesterol (C804518-100mg, Macklin), β-sitosterol (43623-10MG, Supelco), ergosterol (E823641-20mg, Macklin) and stigmasterol (S2424-1G, Sigma-Aldrich).

### Statistical analysis

The results of multiple experiments were presented as the mean ± SD. Data were analyzed via Student’s unpaired t-tests using the GraphPad Prism 8.0 software. Welch’s t-test is used for unequal population variances. Differences were considered statistically significant at p < 0.05.

### Supplementary Information

Below is the link to the electronic supplementary material.

Supplementary file1 (DOCX 3512 kb)

## Data Availability

All data reported in this paper will be shared by the lead contact upon request. Any additional information required to reanalyze the data reported in this paper is available from the lead contact upon request.
