# Supplementary material for: Lipid homeostasis is essential for oogenesis and embryogenesis in the silkworm, Bombyx mori
Source: Cell Mol Life Sci. 2024 Mar 12;81(1):127. doi: 10.1007/s00018-024-05173-8 (PMC10933143; doi:10.1007/s00018-024-05173-8)
Supplement: Supplementary file 1 — Supplementary file1 (DOCX 3512 kb) [file 18_2024_5173_MOESM1_ESM.docx]

**Fig. S1**


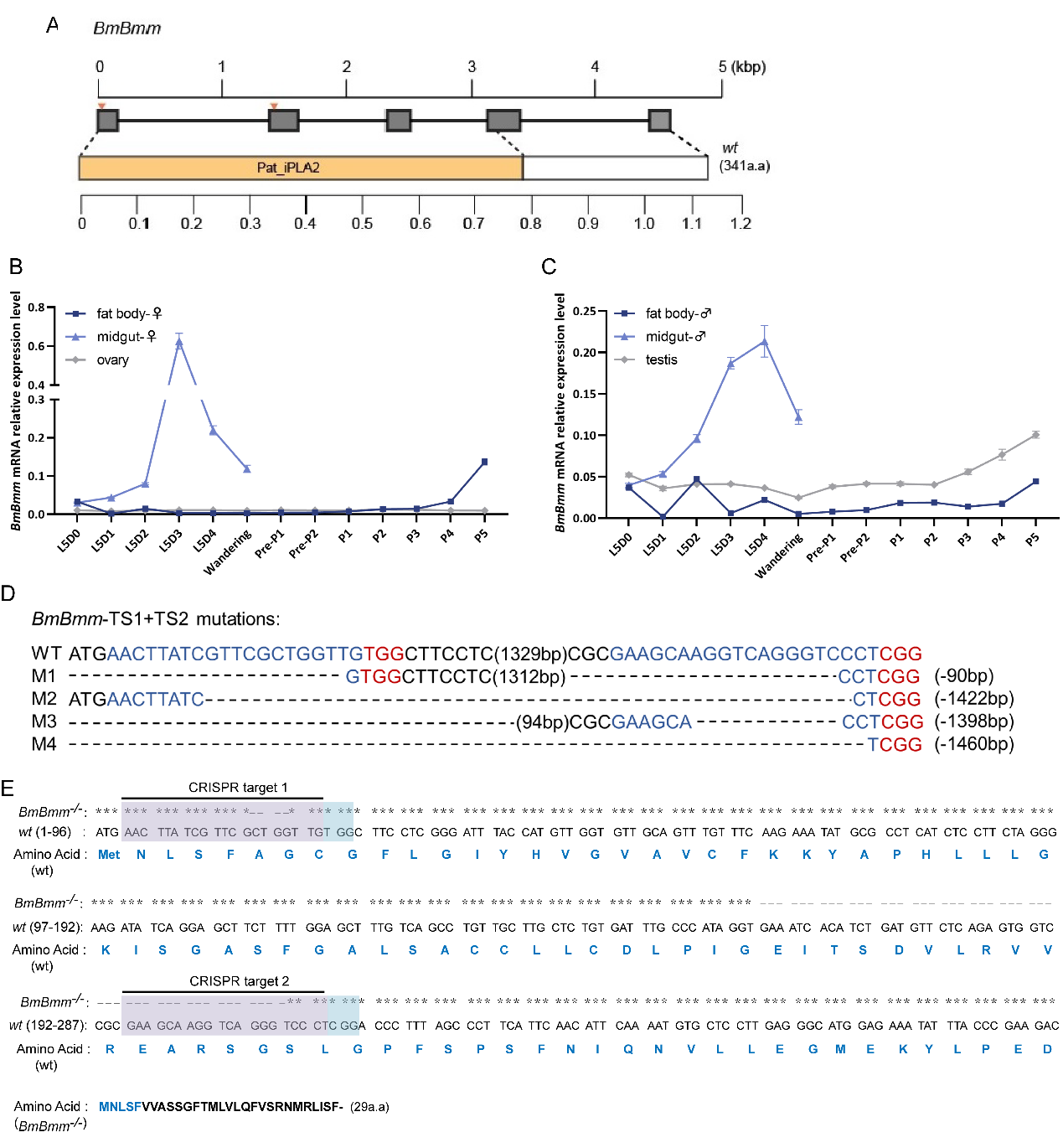


Construction of BmBmm mutants using CRISPR/Cas9 system. **A** Schematic diagram of the gene (top) and protein functional domains (bottom) of *BmBmm*. Gray boxes and solid lines represent exons and introns, respectively. The CDS of *BmBmm* encodes a patatin-like phospholipase domain (Pat_iPLA2) without 5' and 3' untranslated regions. Orange triangles represent sgRNA target sites on the genome. The sizes of mRNA (in bp) are indicated by scale bars. **B** The relative mRNA levels of *BmBmm* were detected every 24 hours from 0 to 240 hours after oviposition and on the first day from 1st to 4th instar. **C and D** The relative mRNA levels of *BmBmm* in fat body, midgut and gonad were detected in females (C) and males (D) at 24 h intervals from the 4th molting (L5D0) to 5th day in pupa (P5). The midgut begins to degenerate at the prepupal stage and is not detected thereafter. **E** Target gene sequences and corresponding encoded amino acids of *BmBmm* deletion homozygous mutant. The sequence of the CRISPR sgRNA target site is indicated by a purple box, and the PAM region is indicated by a blue box. Bases deleted between two target sites of each mutant are indicated by hyphens, and bases identical to wild-type are indicated by an asterisk. *BmBmm^-/-^* encodes a truncated protein containing 29 amino acids.

**Fig. S2**


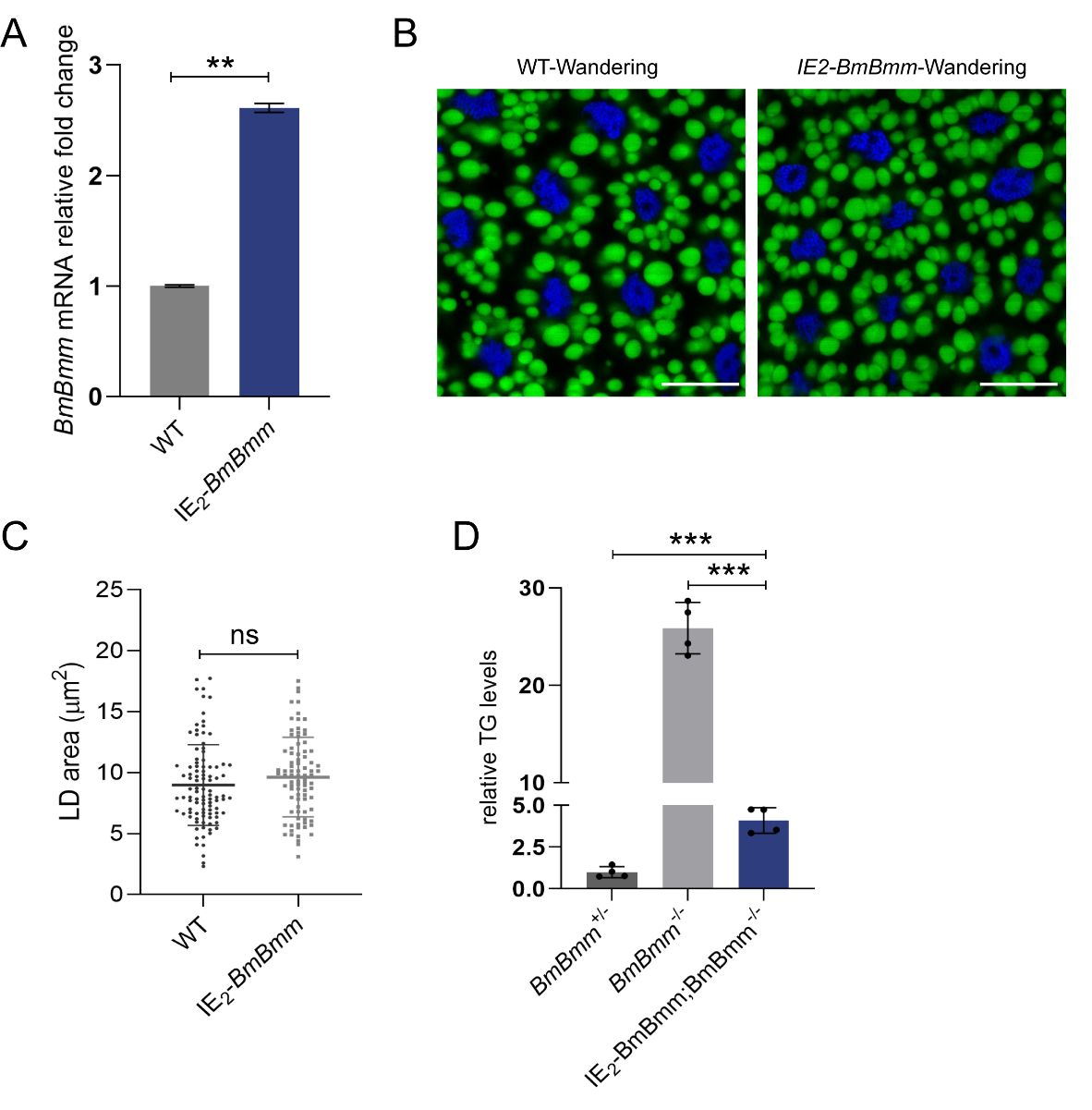


IE_2_-driven *BmBmm* overexpression in *BmBmm^-/-^* alleviates midgut lipid accumulation in *BmBmm^-/-^*. **A** Relative mRNA levels of *BmBmm* in the fat body from WT and IE_2_-*BmBmm* females were determined by qRT-PCR in three biological repeats (each replicate contains three silkworms). Data are normalized to WT. **B** Immunofluorescent staining images of fat body from WT and IE_2_-*BmBmm* larvae at the wandering stage. Cell nuclei were stained with Hoechst, shown in blue; lipid droplets were stained with BODIPY, shown in green. Scale bar represents 15 μm. Quantitative analyses of lipid droplet areas are summarized in (**C**). **D** Relative TG levels in the midgut of female silkworms from *BmBmm*^+/-^, *BmBmm*^-/-^ and IE_2_-*BmBmm*; *BmBmm*^-/-^ (fifth instar, day 2) were determined in four biological replicates (each replicate contains tissue from three animals). Data are normalized to WT. Error bars represent means ± SDs. **p < 0.01; ***p < 0.001; ns: non-significant.

**Fig. S3**


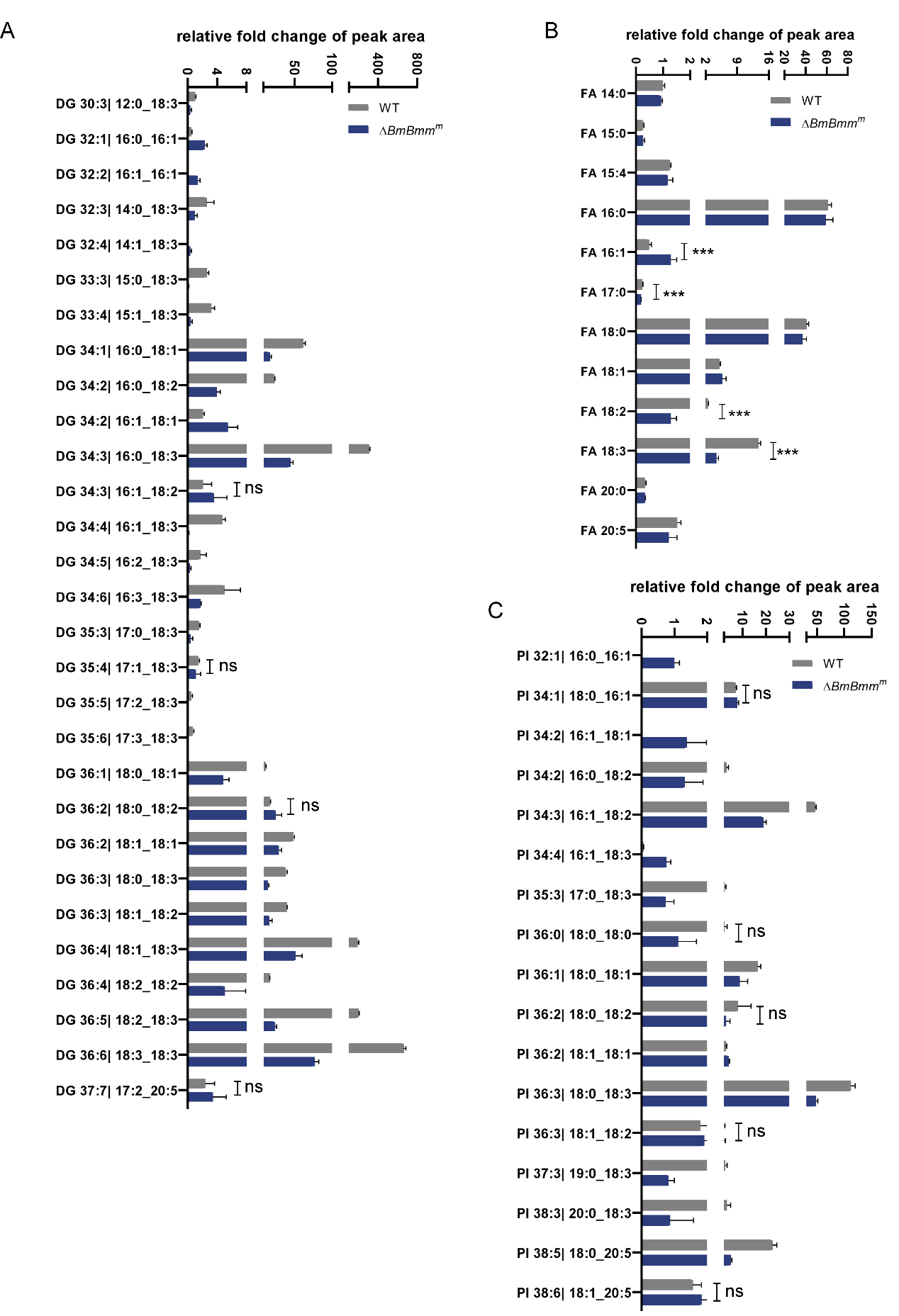


Relative changes of DG, FA and PI contents in the WT and BmBmm^m^. **A** The relative fold change of peak area of diacylglycerol (DG) in the WT and BmBmm^m^ (n=5)**.** ns represents non-significant. The remaining species differ significantly. **B** The relative fold change of peak area of fatty acids (FAs) in the WT and BmBmm^m^. ***p < 0.001. **C** The relative fold change of peak area of phosphatidylinositol (PI) in the WT and BmBmm^m^ (n=5). ns represents non-significant. The remaining species differ significantly. Error bars represent means ± SDs.

**Fig. S4**


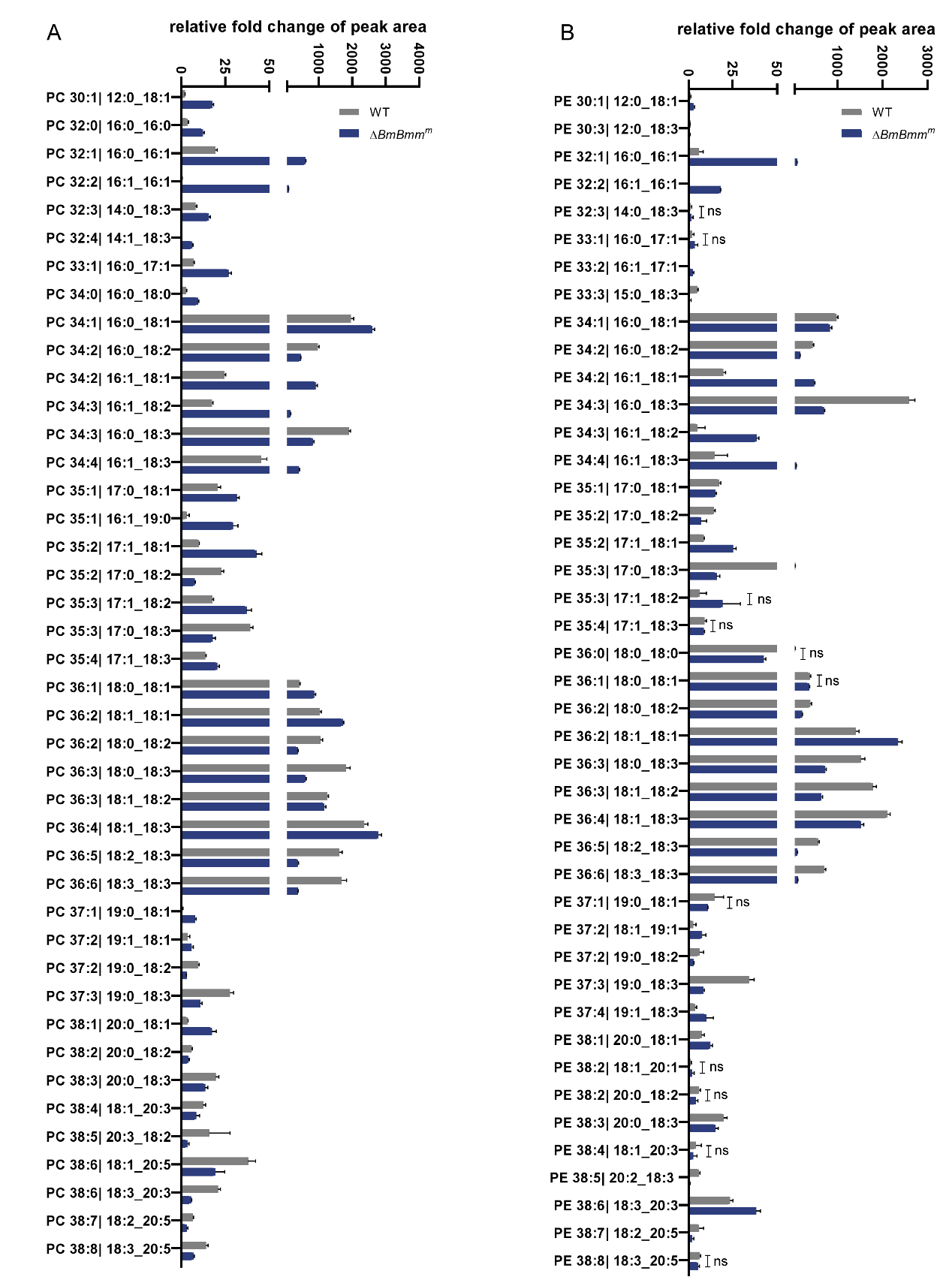


Relative changes of PC and PE contents in the WT and BmBmm^m^. **A** The relative fold change of peak area of phosphatidylcholine (PC) in the WT and BmBmm^m^ (n=5, Student’s t test, p<0.05).  **B** The relative fold change of peak area of phosphatidylethanolamine (PE) in the WT and BmBmm^m^ (n=5). ns represents non-significant. The remaining species differ significantly. Error bars represent means ± SDs.

**Fig. S5**


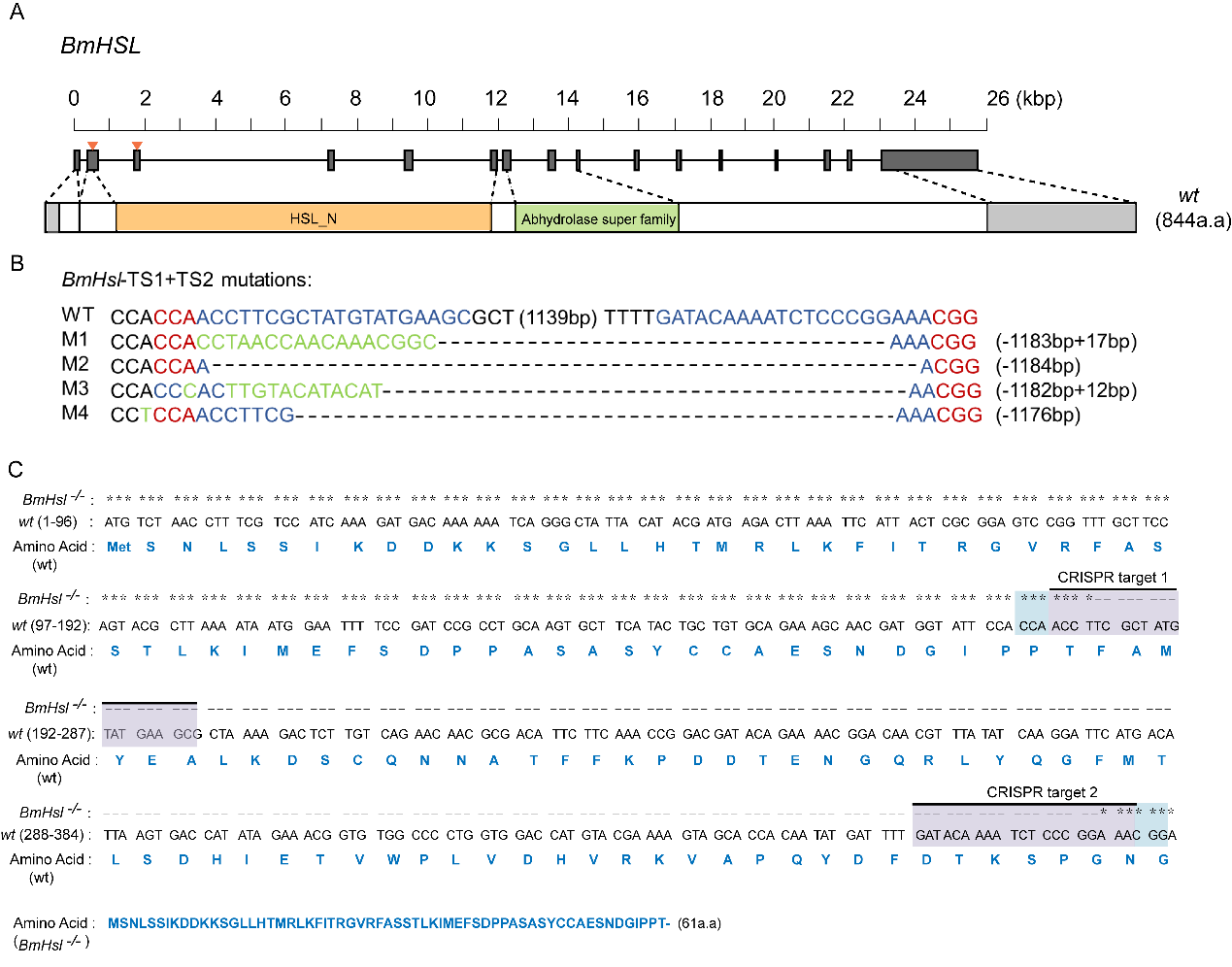


Construction of BmHsl mutants using CRISPR/Cas9 system. **A** Schematic representation of the gene (top) and protein functional domains (bottom) of *BmHsl*. Gray boxes and solid lines represent exons and introns, respectively. The CDS of *BmHsl* encodes the HSL_N domain and the abhydrolase superfamily, carrying 5' and 3' untranslated regions (light gray boxes, left and right, respectively). **B** Base deletions in the target site region of the *BmHsl* mutant. The numbers in parentheses between target sites are the number of bases between the sequences on both sides, the deleted base segment is indicated by a dotted line, and the numbers in parentheses on the right side of each sequence represent the deleted bases. The PAM region is indicated in red font, the target sequence is indicated in blue font, and the inserted base sequence is indicated in green font. **C** Target gene sequences and corresponding encoded amino acids of *BmHsl* deletion homozygous mutant. The sequence of the CRISPR sgRNA target site is indicated by a purple box, and the PAM region is indicated by a blue box. Bases deleted between two target sites of each mutant are indicated by hyphens, and bases identical to wild-type are indicated by an asterisk. BmHsl^-/-^ encodes a truncated protein containing 61 amino acids.

**Fig. S6**


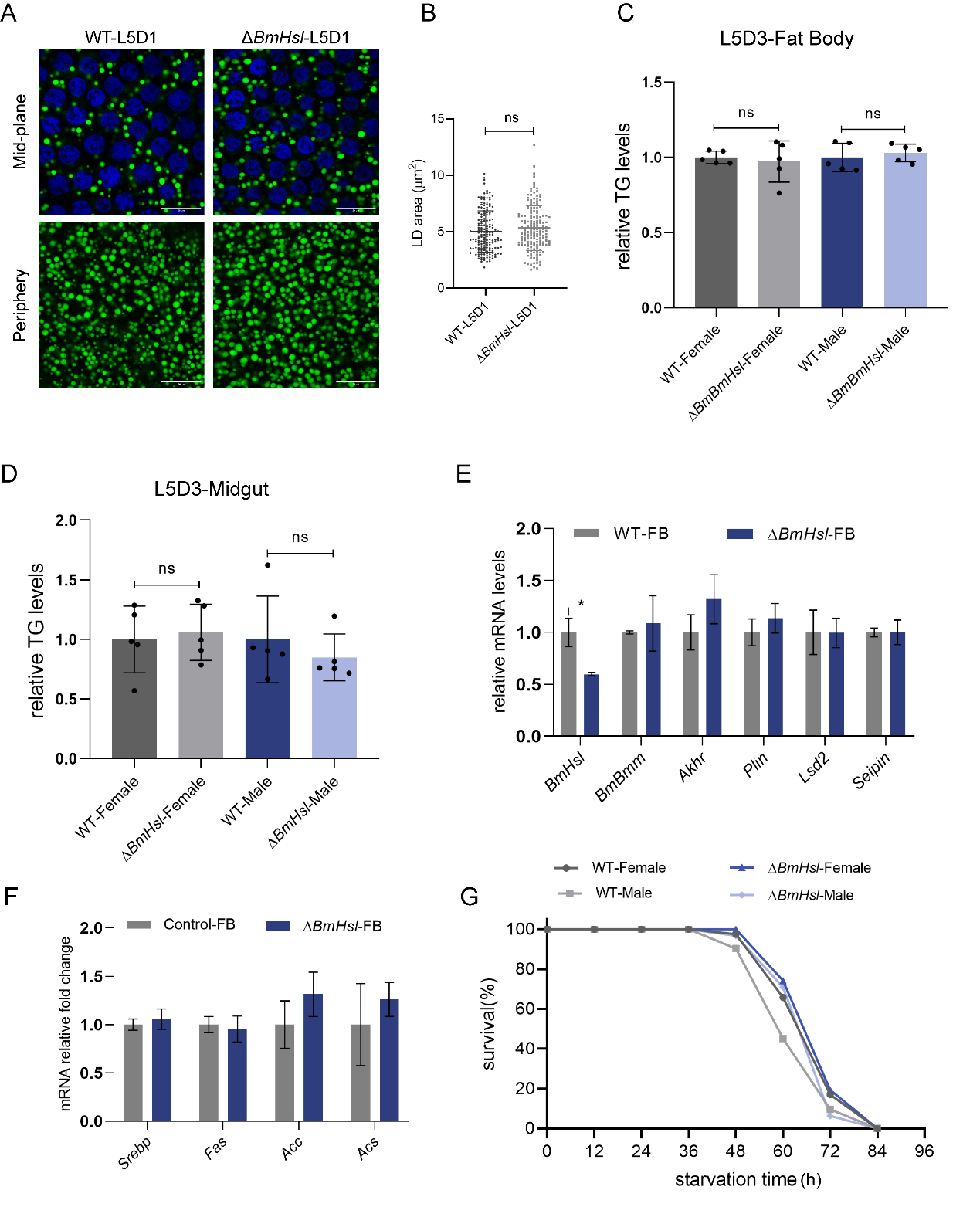


BmHsl deficiency has no significant effect on TG levels and energy mobilization. **A** Immunofluorescent staining of fat bodies. Tissues were obtained from WT and Δ*BmHsl* females on the 1st day of 5th instar. (L5D1). Cell nuclei were stained with Hoechst, shown in blue; lipid droplets were stained with BODIPY, shown in green. Scale bar represents 20 μm. Quantitative analyses of lipid droplet areas are summarized in (**B). C** Relative TG levels in the fat body of female and male silkworms from WT and Δ*BmHsl* (fifth instar, day 3) were determined in five biological replicates (each replicate contains tissue from three animals). Data are normalized to WT.  **D** Relative TG levels in the midgut of female and male silkworms from WT and Δ*BmHsl* (fifth instar, day 3) were determined in five biological replicates (each replicate contains tissue from three animals). Data are normalized to WT. **E and F** Relative mRNA levels of lipolysis (**E**), lipid storage-related (**E**) and fatty acid synthesis (**F**) genes in the fat body from WT and Δ*BmHsl* females were determined by qRT-PCR in three biological repeats (each replicate contains three silkworms). Data are normalized to WT. **G** Survival statistics of larvae under starvation stress conditions. Females and males of WT and Δ*BmHsl* were starved on the 1st day of 5th instar. Error bars represent means ± SDs. *p < 0.05; ns: non-significant.

**Fig. S7**


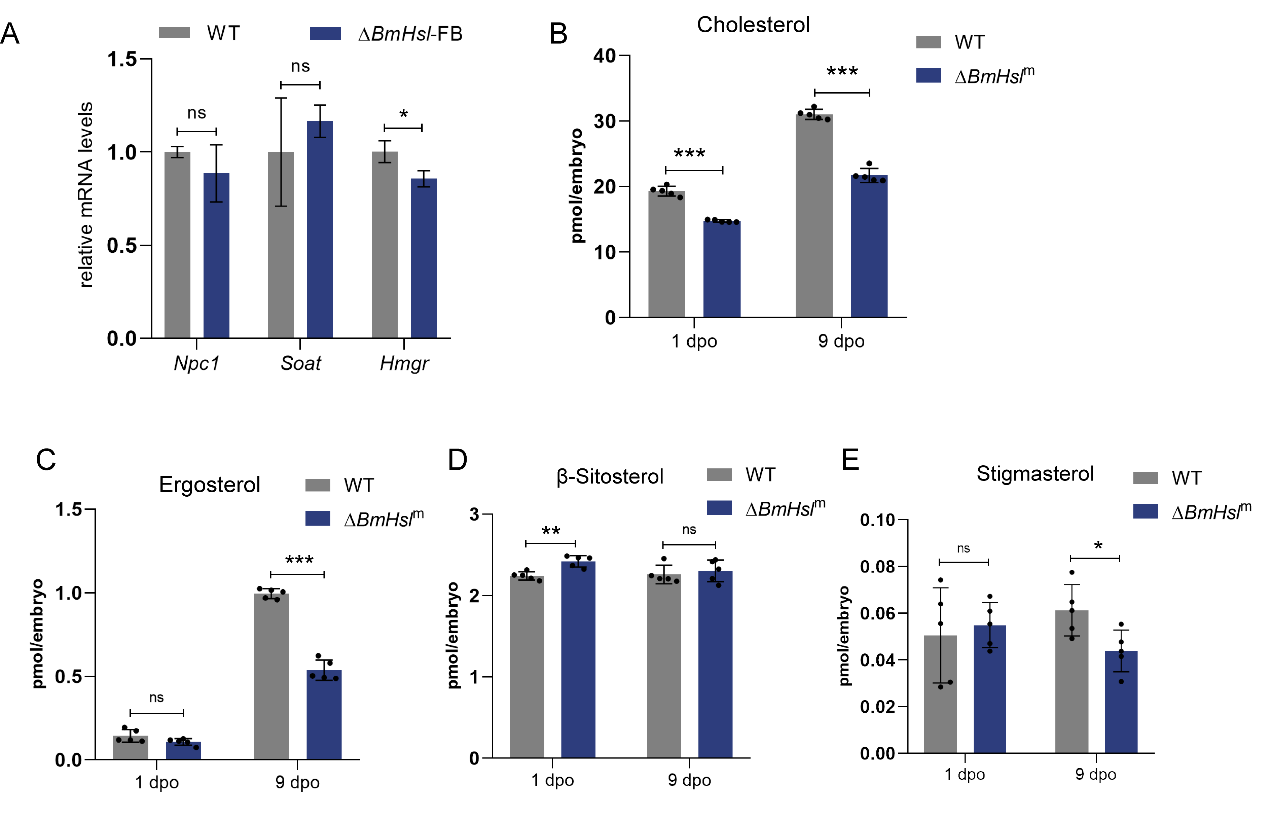


The effect of BmHsl deficiency on the expression of sterol metabolic genes in fat body and the content of free sterols in embryos. **A** Relative mRNA levels of sterol metabolic genes in the fat body from WT and Δ*BmHsl* females were determined by qRT-PCR in three biological repeats (each replicate contains three silkworms). Data are normalized to WT. **B-E** Relative contents of cholesterol (**B**), ergosterol (**C**), β-sitosterol (**D**) and stigmasterol (**E**) in embryos from WT and Δ*BmHsl*^m^ females on the 1st and 9th day post-oviposition (dpo) were determined in five biological replicates (each replicate contains tissue from three animals). Error bars represent means ± SDs. *p < 0.05; **p < 0.01; ***p < 0.001; ns: non-significant.

**Fig. S8**


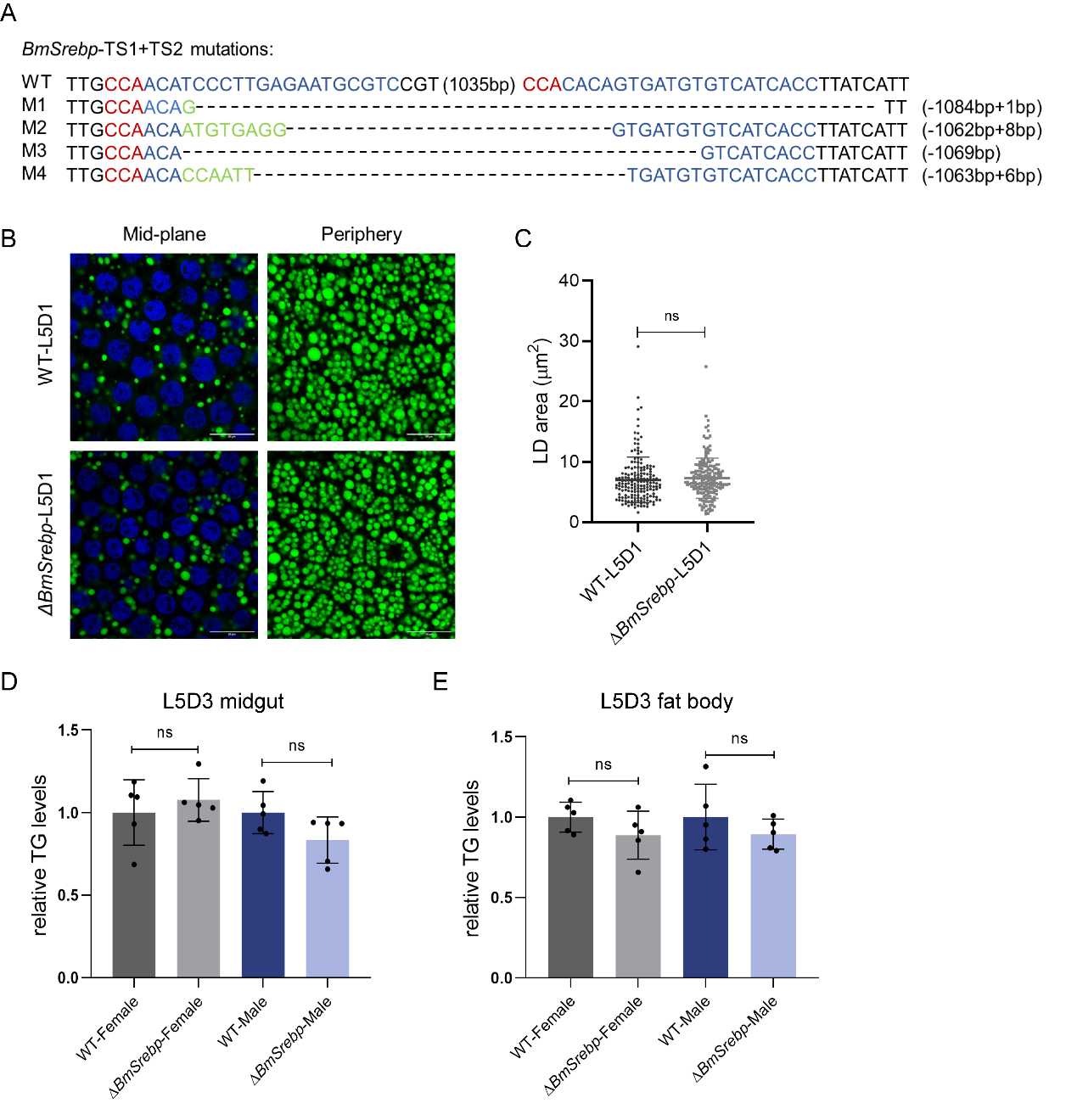


TG levels and energy mobilization are not significantly changed in *BmSrebp* mutants. **A** Base deletions in the target site region of the *BmSrebp* mutant. The numbers in parentheses between target sites are the number of bases between the sequences on both sides, the deleted base segment is indicated by a dotted line, and the numbers in parentheses on the right side of each sequence represent the deleted bases. The PAM region is indicated in red font, the target sequence is indicated in blue font, and the inserted base sequence is indicated in green font. **B** Immunofluorescent staining of fat bodies. Tissues were obtained from WT and Δ*BmSrebp* females (fifth instar, day 1). Cell nuclei were stained with Hoechst, shown in blue; lipid droplets were stained with BODIPY, shown in green. Scale bar represents 20 μm. Quantitative analyses of lipid droplet areas are summarized in (**C**). **D** Relative TG levels in the midgut of female and male silkworms from WT and Δ*BmSrebp* (fifth instar, day 3) were determined in five biological replicates (each replicate contains tissue from three animals). Data are normalized to WT. **E** Relative TG levels in the fat body of female and male silkworms from WT and Δ*BmSrebp* (fifth instar, day 3). Data are normalized to WT. Error bars represent means ± SDs. ns: non-significant.
